# Mitogenomics of historical type specimens of Australasian turtles: clarification of taxonomic confusion and old mitochondrial introgression

**DOI:** 10.1038/s41598-019-42310-x

**Published:** 2019-04-09

**Authors:** Christian Kehlmaier, Xiuwen Zhang, Arthur Georges, Patrick D. Campbell, Scott Thomson, Uwe Fritz

**Affiliations:** 1 Museum of Zoology, Senckenberg Dresden, 01109 Dresden, Germany; 2 Institute for Applied Ecology, University of Canberra, Canberra, ACT 2601 Australia; 3 Department of Life Sciences, Darwin Centre (DC1), Natural History Museum, London, SW7 5BD England UK; 4 Chelonian Research Institute, Oviedo, Florida USA; 5 Museu de Zoologia, Universidade de São Paulo, Avenida Nazaré 481, Ipiranga, São Paulo, SP 04263-000 Brazil

## Abstract

Diagnosability is central to taxonomy as are type specimens which define taxa. New advances in technologies and the discovery of new informative traits must be matched with previous taxonomic decisions based on name-bearing type specimens. Consequently, the challenge of sequencing highly degraded DNA from historical types becomes an inevitability to resolve the very many taxonomic issues arising from, by modern standards, poor historical species descriptions leading to difficulties to assign names to genetic clusters identified from fresh material. Here we apply high-throughput parallel sequencing and sequence baiting to reconstruct the mitogenomes from 18 type specimens of Australasian side-necked turtles (Chelidae). We resolve a number of important issues that have confused the taxonomy of this family, and analyse the mitogenomes of the types and those of fresh material to improve our understanding of the phylogenetic relationships of this morphologically conservative group. Together with previously published nuclear genomic data, our study provides evidence for multiple old mitochondrial introgressions.

## Introduction

Species extinction is occurring at an unprecedented rate^1^, brought about by human-induced habitat loss, overexploitation for economic gain, and climate change. Notwithstanding the urgent need to address the causes of this catastrophic decline in biodiversity, we must understand that many species could be lost before they or their significance have been recognised by science. We are unaware of what it is we are losing. Taxonomic research is arguably more important today than it has been at any time in its history.

Taxonomy has as its foundation the concept of diagnosability. Each new taxon is formally described by identifying one or more traits that consistently distinguish it from other closely related taxa. According to the International Code of Zoological Nomenclature, the name of any new species is unambiguously linked to and defined by the so-called name bearing type specimen. Each generation of taxonomists must deal with new information, brought about by advances in technologies and the discovery of new informative traits. Often they must gather that new information from specimens used by previous generations of taxonomists in making their determinations^2^. Name-bearing type specimens for each named species, and making the types widely available through museums, are thus the foundation of taxonomic enquiry.

DNA technologies have revolutionized taxonomy by bringing to the table a large suite of new characters to complement those available from morphological analysis. At the same time, DNA technology presents a challenge because rarely can the new information be obtained for types used by previous taxonomists. Type specimens are either fixed in formalin, a process that cross-links the DNA with associated proteins and renders the DNA inaccessible, or they are preserved in such a way that DNA is degraded. As a consequence, the accelerating rate at which new biodiversity is being documented is somewhat disconnected from the taxonomic work that has taken place before. Uncertainty over the identity of new forms in comparison to historical type specimens can lead to considerable confusion and taxonomic instability. Generating DNA sequences from historical material has been a major challenge, but the advent of high-throughput parallel sequencing and ancient or trace DNA (aDNA) approaches have fundamentally altered the situation. Although still not routine, generating historical DNA data has become feasible on a large scale^3–6^. DNA sequencing of name-bearing type specimens is thus regarded as the gold standard for taxonomy^7^, because it enables unambiguous assignment of extant populations to the named entity or enables a clear distinction between newly discovered forms and those that have already been described.

Australia is the smallest of all continents and congruent with the nation of the same name. As an island continent, isolated by sea from other vegetated continents for 65 My, it is well known for its endemic fauna and flora. With approximately 755 species, Australia’s reptile diversity exceeds that of any other country for this group^8^. Turtles constitute an iconic group among reptiles that is represented in Australia mostly by the family Chelidae (suborder Pleurodira), side-necked turtles of Gondwanan origin and occurring elsewhere only in New Guinea, on the islands of Roti and Timor of Indonesia and Timor Leste, and in South America^9^. All chelids are freshwater turtles. As the driest vegetated continent on earth, Australia harbours fewer freshwater turtle species than the Americas or Asia. Together with the chelid species from New Guinea, Roti and Timor, there are 36 Australasian species belonging to this family^9,10^. The Australian chelid species often directly compete with humans for water resources and habitat, making their long-term conservation a challenge. For any conservation planning, a sound taxonomy is the necessary prerequisite.

Compared to other continents, the Australasian turtle fauna is poorly known and significant progress has been made only recently^10–21^. Taxonomic assessment of Australasian chelids is impeded by two issues. One is the publication of scientific names in outlets circumventing scientific peer review and contributing to massive confusion by the creation of ill-defined taxa and an alternative competing nomenclature^10,22,23^. The other issue derives from the fact that many historical species descriptions used type specimens without or only with vague locality data, making the identification, and thus the naming, of genetically identified clusters difficult to impossible^14,24^. In addition, the identity of some recently described chelid taxa is unclear (*Chelodina gunaleni*, *C. kuchlingi*, *C. mccordi mccordi*, *C. m. roteensis*, *C. m. timorlestensis*, *C. m. timorensis*) and multiple names could refer to the same taxon^9,10^.

Here we present almost complete mitochondrial genomes for 18 crucial name-bearing type specimens of chelid turtles, and combine this evidence with new mitogenomes of other Australasian chelids and mitogenomes available from GenBank. We use these data to address longstanding controversies arising from uncertainty in the taxonomic identity of historical type specimens. The oldest type specimen sequenced dates back to the late 18^th^ century (*Testudo longicollis* Shaw)^25^. Using whole mitochondrial sequencing, we show the value of generating sequences from historical type specimens in resolving contemporary controversies in taxonomy.

## Materials and Methods

### Sample preparation

Muscle tissue was sampled from the thigh of alcohol-preserved type specimens (Table S1); tissue from dry specimens, from inside the shell or the inguinal region of the shell. Fresh material (Table S2) was obtained from turtles sampled alive by cutting a sliver of skin from the trailing web of the hind foot or by sampling blood from the jugular vein in accordance with the “*Standard Operating Procedures (SOPs) for the care and use of animals in laboratory studies, teaching and field research*” developed by the Animal Ethics Committee of the University of Canberra. We did not conduct experiments on the live turtles, but the procedures used to sample tissue and blood were nevertheless subject to the SOP. Thus, the samples were obtained according to all relevant guidelines, regulations and best ethical and experimental practice of the University of Canberra. The blood and tissue samples were collected as part of other studies; the samples for this publication were accessed from the Wildlife Tissue Collection of the University of Canberra. No samples were specifically collected from live animals for this study.

Mitochondrial genomes for the live specimens were generated as described in Zhang *et al*.^26^. Mitochondrial genomes for the type specimens were generated by extracting DNA using commercial DNA extraction kits. Illumina libraries were prepared according to Meyer & Kircher^27^ as modified by Fortes & Paijmans^28^; preparing bait libraries and conducting two rounds of in-solution hybridization capture following the methods of Maricic *et al*.^29^ and Horn^30^; and sequencing on an Illumina MiSeq sequencing platform. Detailed methods are described in the Supplementary Information. To avoid cross-contamination, samples were divided into categories of historical samples and recent samples, and processed in laboratories that were physically separated. DNA extractions and Illumina library preparation for the historical samples (13 samples collected between ~1790–1990) were undertaken in the clean room facility of Senckenberg Dresden, which meets all standards of an ancient DNA laboratory as outlined in Fulton^31^. Fresh materials (6 samples collected between 1994–2007) were processed in the main molecular laboratory of Senckenberg Dresden. Negative controls (water blanks) were included during DNA extraction and library preparation and screened for evidence of contamination after sequencing. The remaining fresh samples were processed in the PC2 facilities at the Wildlife Genetics Laboratory at the University of Canberra.

### Assembly

Demultiplexing the sequence reads and generating of the fastQ files were automatically performed by the miseq reporter 2.6.2.3. Subsequently, adapters were trimmed with skewer 0.2.2^32^, reads merged (minimum read length 35 bp) and duplicates removed with BBMap-suite 37.24 (https://sourceforge.net/projects/bbmap/)^33^. Sequence length distribution of merged single reads was checked using a customized command written in awk. Subsequent visualisation used microsoft excel. Subsamples from the full readpool were created with fastq-tools v 0.8 (https://github.com/dcjones/fastq-tools) to keep the average read coverage below 200x. Assembly used the MITObim pipeline^34^, which uses MIRA4^35^ in a two-step baiting and iterative mapping. The complete mitochondrial sequence of KJ713173 (*Chelodina longicollis*) was used as a starting seed. For each sample, three different mapping stringencies were tested, setting the allowed mismatches to 15% of the average read length (MM15, default value), to a maximum of five (MM5), and to a maximum of two (MM2). Sequence length distribution of the assembled merged reads was obtained as described above. A visual exploration of the resulting assemblies for assembly artefacts and regions of conflicts (mostly low coverage regions) was performed using tablet 1.16.09.06^36^. For each sample, the obtained contigs (the assembled mt-genome sequences) of the MM15 and the MM2 analyses were manually compared. If they were identical, the assembled mt-sequence was accepted. For those samples where differences occurred and obvious assembly artefacts could not be corrected by hand, a consensus sequence of the corresponding MM2 and MM5 analysis was obtained, masking all conflicting positions with Ns (ambiguous nucleotide positions). After the assembly, PCR priming sites at the 5′ and 3′ ends of the assembled mt-sequences were removed before submitting them to a BLAST search.

Each library was also analysed using the complete mitochondrial sequence of KU867578 (*Homo sapiens*) as a starting seed to check for human contamination.

### Sequence alignment and annotation

Each mt-sequence was first annotated under default parameters using mitos^37^ to check gene arrangement. Then an automated pre-alignment of all sequence data was conducted using clustalw 1.4 as implemented in bioedit 7.0.9.0^38^, with default parameters^39^. A final alignment adjustment and sequence annotation was performed manually, based on four published mt-genomes: AF039066 (*Pelomedusa*), KY486272 (*Pseudemydura*), KC692462 (*Emydura*), and KJ713173 (*Chelodina*). Two alignments were produced, one based on full sequences (FULL), the second on coding sequences only (CDS). Further details of the alignment and annotation are provided in the Supplementary Information.

### Phylogenetic analysis and genetic distances

Phylogenetic relationships of the mitogenomes were estimated by applying RAxML 8.0.0^40^ for Maximum Likelihood (ML), and mrbayes 3.2.6^41^ for Bayesian inference (BI), to the CDS and FULL alignments. The selection of the best fitting evolutionary models (Tables S3, S4) and the data partitioning schemes for the analyses was computed with partitionfinder 2^42^ using the Bayesian Information Criterion. Five different partition schemes were considered (unpartitioned—1 partition; third codon position extra, i.e., first and second codon positions combined, third codon position extra, 12 S, 16 S, control region, all tRNAs combined—6 partitions; each codon position extra, i.e., as preceding partitioning scheme except first and second codon positions extra—7 partitions; gene-partitioned, i.e., 13 coding genes, 12 S, 16 S, control region, tRNAs combined—17 partitions; codon-partitioned, i.e. 13 times 3 codon positions extra, 12 S, 16 S, control region, tRNAs combined—43 partitions). For ML and BI, the codon-partitioned dataset was selected. For ML, five independent rapid bootstrap searches were run using different starting conditions. Subsequently, 1000 non-parametric thorough bootstrap replicates were calculated and the values plotted against the best tree. Two parallel runs (each with four chains) were performed for BI for 10 million generations (burn-in fraction 0.25; print frequency 1000; sample frequency 500). The calculation parameters were analysed using the software tracer 1.7.1^43^. Posterior probabilities were plotted onto the phylograms. MEGA7^44^ was used for exploratory calculations of uncorrected *p* distances using the pairwise deletion option.

## Results

In total, 52 mitochondrial genomes were analysed, including one outgroup (*Pelomedusa*), of which 15 were taken from GenBank and 37 (including 18 types) were newly generated (Tables S1, S2). Among the historical type specimens, only processing of the holotype of *Platemys novaeguineae* Meyer, 1874 failed. This specimen was boiled during the firestorm of Dresden following allied bombing in the Second World War^45^. Assembly information of the sequenced type specimens such as size of readpool, number of assembled reads, average coverage, and average read length of assembled reads are summarized in the Supplementary Information. The aligned data covered almost the entire mitochondrial genome, missing only part of the control region and part of the DNA coding for tRNA Phe. All samples had a typical vertebrate mitochondrial genome organisation^46^. For further details of the alignment (e. g., observed frame shifts in coding regions), see Supplementary Information.

### Phylogeny and species assignment

**Figure 1:**
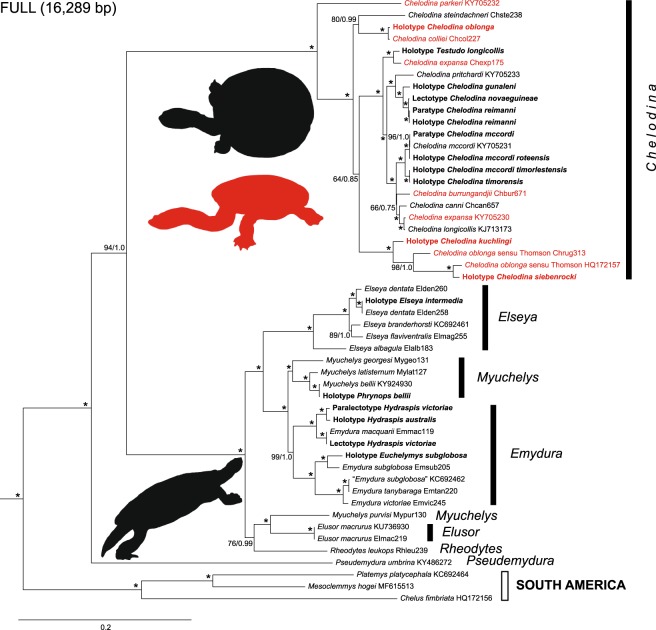
Maximum Likelihood tree for chelid mitogenomes, including historical type specimens using a 16,289-bp-long alignment. For historical type specimens, the original name combinations are shown in bold. Numbers at nodes are bootstrap values and posterior probabilities for a Bayesian tree of the same topology. Asterisks indicate maximum support under both methods. Quotation marks for a GenBank sequence assigned to *Emydura subglobosa* (KC692462) indicate questionable taxonomic allocation. Lectotype and paralectotype of *Hydraspis victoriae* were previously erroneously identified. The species description was based only on the putative paralectotype, which is therefore the name-bearing holotype (see Discussion). Snake-necked species of *Chelodina* in red, long-necked species in black. Icons are derived from photos of *Chelodina steindachneri* (long-necked *Chelodina*), *C. burrungandjii* (snake-necked *Chelodina*), and *Elusor macrurus* (short-necked species; from top to bottom). Drawings: U. Fritz. On the right are for Australasian taxa the currently accepted genera shown^9^.

**Figure 2:**
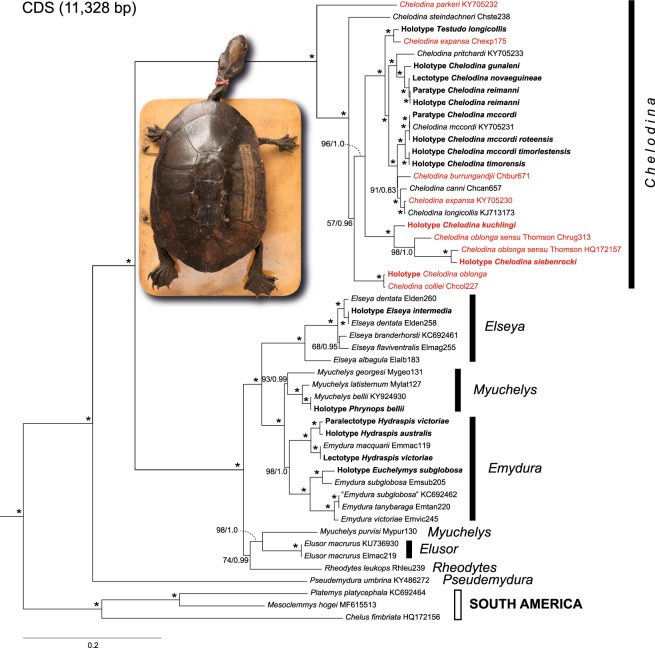
Maximum Likelihood tree for chelids, including historical type specimens using an 11,328-bp-long alignment corresponding to the 13 coding genes of the mitogenome. For historical type specimens, the original name combinations are shown in bold. Quotation marks for a GenBank sequence assigned to *Emydura subglobosa* (KC692462) indicate questionable taxonomic allocation. Numbers at nodes are bootstrap values and posterior probabilities for a Bayesian tree of the same topology. Asterisks indicate maximum support under both methods. Note the different placement of *Chelodina steindachneri* compared to Fig. 1. Snake-necked species of *Chelodina* in red, long-necked species in black. On the right are for Australasian taxa the currently accepted genera shown^9^. Inset: historical type specimen of *Testudo longicollis* Shaw, 1794 (photo: P. D. Campbell). For further explanation, see Fig. 1.

The Maximum Likelihood (ML) and Bayesian inference (BI) trees for each dataset were highly consistent, with identical branching patterns and similar support values (Figs 1 and 2). Most nodes had maximum support under both tree-building methods. Placement of only one taxon, *Chelodina steindachneri*, differed between the phylogenies generated from the two alignments (‘FULL’ and ‘CDS’).

South American and Australasian chelids were revealed as maximally supported sister clades. Within the Australasian taxa, *Pseudemydura umbrina* constituted the deeply divergent sister taxon of two other deeply divergent, more inclusive clades. One of these consisted of the snake-necked and long-necked turtles of the genus *Chelodina* and the other comprised short-necked turtles of the genera *Elseya*, *Elusor*, *Myuchelys*, and *Rheodytes*. Many of the nominal taxa within *Chelodina* showed smaller genetic divergences than the taxa within the ‘short-necked clade’.

Within the snake-necked and long-necked turtle clade, *C. parkeri* represented the deeply divergent sister taxon of all remaining nominal *Chelodina* species (Figs 1 and 2). Using coding sequences (CDS dataset; Fig. 2), the deeply divergent *C. steindachneri* and a clade comprising the holotype of *C. oblonga* plus a fresh sample of *C. colliei* represented the successive sister taxa of two crown clades including the remaining nominal *Chelodina* taxa. However, using the FULL dataset (Fig. 1), *C. steindachneri* clustered with moderate support with the clade containing the *C. oblonga* type and *C. colliei*. In any case, the sequences of the holotype of *C. oblonga* and the fresh sample of *C. colliei* were nearly identical, and together distinct.

One of the two crown clades of *Chelodina* showed substantial genetic structure and contained as successive sister taxa the holotype of *C. kuchlingi*, a fresh sample of *C. oblonga* sensu Thomson^24^ (Finnis River, Northern Territory) and a terminal clade comprised of the holotype of *C. siebenrocki* plus a GenBank mitogenome of *C. oblonga* sensu Thomson^24^ of unknown provenance. Both the holotype of *C. kuchlingi* and the *C. oblonga* sensu Thomson^24^ from the Finnis River were clearly distinct with long branches, whilst branch lengths for the holotype of *C. siebenrocki*, a nominal species described from New Guinea, and the GenBank mitogenome of *C. oblonga* sensu Thomson^24^ were only moderately differentiated. The other crown clade contained three clades with individual terminals having short branches. One terminal clade contained the holotype of *Testudo longicollis* plus a fresh sample of *C. expansa* from the Murray-Darling drainage. The second terminal clade consisted of taxa from New Guinea, with *C. pritchardi* being sister to the holotype of *C. gunaleni* plus a virtually undifferentiated clade containing the lectotype of *C. novaeguineae* and the holotype and a paratype of *C. reimanni*. The third terminal clade comprised taxa from Australia and the islands of Roti and Timor. The paratype of *C. mccordi* and a fresh sample of the same species were virtually undifferentiated from the holotype of *C. mccordi roteensis*. The sequences for the holotypes of *C. mccordi timorlestensis* and *C. timorensis* were identical and sister to the aforementioned clade containing among others the paratype of *C. mccordi*. The remaining sequences from the same terminal clade corresponded to Australian taxa, constituting a weakly supported clade with *C. burrungandjii* being sister to a well-supported clade embracing *C. canni* as sister taxon of the weakly differentiated sequences from a *C. expansa* from the Dawson River, Fitzroy, Queensland (GenBank accession number KY705230) and a *C. longicollis* from the Australian Capital Territory^47^ (KJ713173).

Within the short-necked clade, a moderately supported clade embracing *Rheodytes leukops*, *Elusor macrurus*, and *Myuchelys purvisi* was sister to the remaining taxa. *Elusor macrurus* and *M. purvisi* were in this clade as maximally supported sister taxa, rendering *Myuchelys* polyphyletic. Among the remaining taxa, the species of the genus *Elseya* were monophyletic and sister to two other clades, one containing the species of *Emydura* and the other those of *Myuchelys*, except *M. purvisi*. With respect to the studied type specimens, the sequence of the holotype of *Elseya intermedia* was identical with a sequence of *El. dentata* from the Victoria River, Northern Territory (Elden258), and the sequence of the holotype of *Phrynops bellii* was identical to that of a fresh sample of the same species (*M. bellii*) from the Gwydir River, Murray-Darling Basin, New South Wales. Within *Emydura*, the sequence of the lectotype of *Hydraspis victoriae* was identical to a sequence of a fresh sample of *Em. macquarii* from the Murray River, Murray-Darling Basin, whilst the paralectotype of *Hydraspis victoriae* clustered with maximum support with the holotype of *Hydraspis australis*. Both types of *Hydraspis victoriae* did not cluster with a fresh sample identified with *Em. victoriae*. This fresh sample of *Em. victoriae* from the Daly River, Northern Territory, was placed together with a GenBank sequence of *Em. subglobosa* (KC692462) of unknown provenance and *Em. tanybaraga* from the Mitchell River, Queensland, in a maximally supported clade which was sister to a clade comprising a fresh sample of *Em. subglobosa* from Roper River, Northern Territory, and the sequence of the holotype of *Euchelymys subglobosa*. The latter two sequences clustered with maximum support, but their genetic divergence resembled those between *El. branderhorsti* and *El. flaviventralis* and exceeded the divergence between *Em. tanybaraga* and a fresh sample of *Em. victoriae* from the Daly River, Northern Territory.

## Discussion

Our analysis of mitogenomes, including those of key type specimens, resolves a number of taxonomic and nomenclatural controversies. Perhaps the most significant is that of the status of *Chelodina oblonga* Gray, 1841, which has been controversial for 130 years^48^. The near-identity of the mitogenomes of the holotype of *C. oblonga* and what is currently named *C. colliei* Gray, 1856 challenge the nomenclatural conclusions of Thomson^24,48^. Using morphological evidence, this author identified *C. oblonga* as conspecific with what was before *C. rugosa* Ogilby, 1890, rendering the latter name a junior synonym of *C. oblonga*, whilst for the southwestern species previously identified with *C. oblonga* the name *C. colliei* had to be reinstated. Considering this contorted nomenclatural history and how dubious the holotype of *C. oblonga* has become, with disagreement between molecular data (this study), collection data, and morphology^24,48^, we declare *C. oblonga* Gray, 1841 a *nomen dubium*. This delivers nomenclatural stability to this group of species, with *C. colliei* applying to the long-necked turtle confined to the southwest of Western Australia. For the species that was since Thomson^48^ identified with *C. oblonga*, the name *C. rugosa* Ogilby, 1890 has to be resurrected.

Within this species complex, *C. rugosa* Ogilby, 1890 and *C. siebenrocki* Werner, 1901 have been variously regarded as distinct species^49–52^ or the same^53–55^. Morphological evidence in support of their separation is scant. Burbidge *et al*.^51^ (p. 393) mention only ‘consistent, if minor, differences’ between the two as their basis for recognizing them as separate species. Rhodin & Mittermeier^56^ failed to reliably distinguish *C. rugosa* and *C. siebenrocki* following a morphological analysis, and chose to refer to them collectively as the *C. rugosa* complex. In the absence of any substantive diagnostic morphological differences and any fixed allozyme differences, Georges *et al*.^57^ synonymised *C. siebenrocki* with *C. rugosa*. The mitogenome of the holotype of *C. siebenrocki* is weakly differentiated (1.51%) from the mitogenome of a *C. rugosa* of unknown origin (GenBank HQ172157; Figs 1 and 2). However, their divergence is similar to that of other *Chelodina* species or subspecies (*C. gunaleni* vs. *C. novaeguineae* and *C. reimanni*: 1.37–1.41%; *C. m. mccordi* vs. *C. m. timorensis* as defined below: 1.01–1.04%; *C. canni* vs. the sister clade comprised of introgressed *C. expansa* and *C. longicollis*: 1.33–1.40%; Table S5). Another mitogenome of *C. rugosa* from the Finnis River, Northern Territory (sample Chrug313), is deeply divergent from both and differs by 5.53% and 5.71% uncorrected *p* distance from the holotype of *C. siebenrocki* and the GenBank mito-genome of *C. rugosa*, respectively, suggesting that more than one species is involved here. This is also supported by the previously reported deep divergence between two samples of *C. rugosa* from Queensland and the Northern Territory using 45 allozyme loci^57^. For the Northern Territory form the name *Chelodina intergularis* Fry, 1915 is available, if it should be considered to represent a separate species in the future.

The sister taxon of the clade comprising the mitogenomes of *C. rugosa* (= *C. oblonga* sensu Thomson) and *C. siebenrocki* is represented by the mitogenome of the holotype of *C. kuchlingi* Cann, 1997. This nominal species has been regarded variously as a valid taxon^9,14,58^ or not^10,59^ because it was described from a single specimen of uncertain provenance, without a scientifically defensible diagnosis or description. No living specimens of *C. kuchlingi* have been found since its description, despite extensive searches. With respect to uncorrected *p* distances, the mitogenome of the *C. kuchlingi* type differs from that of the most similar species, a *C. rugosa* from the Finnis River, Northern Territory (sample Chrug313), approximately 660 km east of the type locality of *C. kuchlingi*, by 3.78% and from other *Chelodina* species, by 5.72–10.37% (Table S5). Considering only the COI gene, we found that *C. kuchlingi* differs from *C. rugosa* and *C. burrungandjii* by between 4.4% and 6.4%. This is in contrast to a previous report, without supporting data or a clear indication of the specimens sequenced, that *C. kuchlingi* differs by 14–18% in mitochondrial COI sequence from other species of northern snake-necked turtles (*C. oblonga* = *C. rugosa* and *C. burrungandjii*)^58^. Nevertheless, our data support *C. kuchlingi* as a valid species. However, the stated type locality of Kalumbaru^60^ is in doubt^10^, and we were unable to link the taxon to any population of *Chelodina*. Locating populations of this species is thus a high priority.

Our analysis also resolves the confusion over taxa associated with the name *Chelodina mccordi* Rhodin, 1994. Two taxa have been recognised from the island of Roti, namely *Chelodina mccordi mccordi* Rhodin, 1994 from the western side of the island and *Chelodina mccordi roteensis* McCord, Joseph-Ouni & Hagen, 2007 from the eastern side^9^. The mitogenomes of the holotype of *C. m. roteensis* and a topotypic paratype of *C. mccordi* (and of a non-type sample of *C. mccordi* from Roti) are identical. We therefore formally place *C. m. roteensis* McCord, Joseph-Ouni & Hagen, 2007 in the synonymy of *C. m. mccordi* Rhodin, 1994.

The mitogenomes of the turtles from Roti island are slightly distinct from and sister to those of the holotypes of *C. m. timorensis* McCord, Joseph-Ouni & Hagen, 2007 and *C. m. timorlestensis* Kuchling, Rhodin, Ibarrondo & Trainor, 2007 from Timor Leste. The mitogenomes of the holotypes of *C. m. timorensis* and *C. m. timorlestensis* are identical, matching their current understanding as one and the same taxon^9^.

With respect to the *Chelodina* species from New Guinea, the mitogenome of *C. pritchardi* differs from that of other *Chelodina* species by uncorrected *p* distances between 3.1% (*Chelodina novaeguineae* and related taxa) and 11.04% (*C. parkeri*), supporting its distinctness. The mitogenomes of the holotype and paratype of *Chelodina reimanni* Philippen & Großmann, 1990, however, show negligible divergence from that of the lectotype of *C. novaeguineae* Boulenger, 1888 (0.2%). Using 45 allozyme loci, the two taxa were also found undifferentiated^57^. This suggests that the distinctive morphology of *C. reimanni* represents phenotypic plasticity, perhaps in response to a food source that stimulates macrocephaly. Macrocephaly is also known from other turtle species and thought to represent an adaptation to crushing hard-shelled food. Besides generally broad-headed species^61^, macrocephaly also occurs in some taxa only locally or as a sex-specific trait (Chelidae: *Emydura australis, E. krefftii*, *E. tanybaraga*, *E*. *victoriae*, *E. subglobosa worrelli*^58^; Emydidae: *Emys orbicularis hellenica*^62^; *Graptemys barbouri*, *G. ernsti*, *G. gibbonsi*, *G. pearlensis*, *G. pulchra*^63,64^; Geoemydidae: *Mauremys reevesii*^61^; Trionychidae: *Apalone ferox*^65^; for further examples, see Iverson *et al*.^61^), supporting that *C. reimanni* could be only a local variant of *C. novaeguineae*. Alternative explanations for the near-identity of their mitogenomes and the lack of differentiation in allozymes are mitochondrial introgression or recent origin of both taxa. We tentatively recognize *C. novaeguineae* and *C. reimanni* as distinct taxa pending further research using additional morphological data and nuclear markers applied across a more comprehensive geographical range of the species.

The status of *C. gunaleni* McCord & Joseph-Ouni, 2007 from New Guinea has also been controversial^9,10,66,67^, and Georges & Thomson^10^ placed *C. gunaleni* into the synonymy of *C. novaeguineae*. In our mitogenomic phylogeny, *C. gunaleni* is the sister taxon of *C. novaeguineae*/*C. reimanni* (Figs 1 and 2). The mitogenome of the holotype of *C. gunaleni* differs by uncorrected *p* distances of 1.37–1.41% from the mitogenomes of *C. novaeguineae* and *C. reimanni* (Table S5), supporting the proposed validity of *C. gunaleni*^9^.

For the short-necked species, we are able to support the identification of the holotype of *Phrynops bellii* Gray, 1844 of unknown provenance with the *Myuchelys* species from the Gwydir and Namoi Rivers of the Murray-Darling Basin, as suggested by Cann^14^. This author investigated the itineraries of potential collectors of the historical type specimen and concluded that it came from west of the Great Dividing Range, and could not represent *Myuchelys latisternum* to which it might otherwise have been assigned. Our mitogenome data complements this circumstantial evidence and confirms the identity of *Myuchelys bellii* (Gray, 1844).

Furthermore, we were able to confirm the synonymy of *Elseya intermedia* Gray, 1872 with *Elseya dentata* (Gray, 1863). The mitogenome of *E. intermedia* is virtually identical to a fresh sample of *E. dentata* from the type locality of Victoria River, Northern Territory (Elden258), supporting the view that *E. intermedia* Gray, 1872 is a junior synonym of *Chelymys dentata* Gray, 1863^9,10,52^.

The mitogenomes of the other type specimens of short-necked chelids are relevant to some complicated taxonomic issues. In a much-debated privately published document (see the review in Iverson *et al*.^68^), Wells & Wellington^69^ designated in 1985 a lectotype (BMNH 1947.3.5.95) for *Hydraspis victoriae* Gray, 1842, assuming that the original description of this species^70^ was founded on two specimens. Using morphology, Iverson *et al*.^68^ concluded that these two putative type specimens represent two distinct species. The name-bearing lectotype was identified as the species currently called *Emydura macquarii*, rendering *Hydraspis victoriae* Gray, 1842 a junior synonym of *Chelys (Hydraspis) macquarii* Gray, 1830, whilst the paralectotype of *H. victoriae* (BMNH 1947.3.5.96) is an *Em. victoriae* following the intent of its describer Gray^70^. Our results support that the lectotype is an *Em. macquarii*. The mitogenome of the lectotype differs from that of an *Em. macquarii* from the Murray River, New South Wales (Emmac119), by only three mutations (0.02% uncorrected *p* distance; Table S5). The mitogenome of the paralectotype of *H. victoriae* is distinct and clusters with that of the holotype of *H. australis* Gray, 1841, with 0.68% uncorrected *p* distance between both. These two mitogenomes together are sister to *Em. macquarii*, represented by the individual from the Murray River and the lectotype of *H. victoriae* (2.31–2.35%; Table S5), providing evidence against their identity. This intricate situation is further complicated by the occurrence of a third mitogenome from a turtle identified with *Em. victoriae* from the Daly River, Northern Territory (Emvic245) in another deeply divergent clade, clustering together with the mitogenomes of *Em. subglobosa* and *Em. tanybaraga* (Figs 1 and 2). This suggests that the turtles from the Daly River identified with *Em. victoriae* represent another species, as supported by previously published allozyme evidence and distinct coloration^71^.

The nomenclatural situation is further confused by the fact that Cann & Sadlier^58^ concluded recently that the superficial description of *Em. victoriae* (Gray, 1842) was based on only one individual (in their view BMNH 1947.3.5.96), so that the type status of one of the two currently recognized type specimens is challenged. Gray’s description of *Hydraspis victoriae*^70^ gave no indication how many specimens were available and his intention was clearly to describe a new species from the Victoria River, Northern Territory. As such, the specimen selected by Wells & Wellington^69^ as lectotype (BMNH 1947.3.5.95), most likely from the Murray River in southern Australia, cannot be considered a type specimen of *Hydraspis victoriae* even though it has been regarded as such since the late 19^th^ century^72^. In addition, it was associated with erroneous locality data (“Victoria River, N. W. Australia”) ever since this was stated in Boulenger’s *Catalogue of the Chelonians, Rynchocephalians, and Crocodiles in the British Museum (Natural History)* in 1889^72^. However, the handwritten question mark after the locality in Boulenger’s private copy of the *Catalogue* in the Natural History Museum, London, shows that Boulenger was in doubt about the provenance of this specimen. That BMNH 1947.3.5.95 is not a type of *Hydraspis victoriae* is further corroborated by the observation that Gray^73^ (p. 42) earlier explicitly mentioned only one specimen in his *Catalogue of the Tortoises, Crocodiles, and Amphibaenians in the Collection of the British Museum* of 1844, a shell, identified with “*H. Victoriae*, Gray” from the “Victoria River, N. W. coast of Australia”. This is clear evidence that the original description of *Hydraspis victoriae* Gray, 1842 was based on only one specimen (BMNH 1947.3.5.96), which has to be regarded as the holotype, making the previous designation of BMNH 1947.3.5.95 as lectotype^69^ invalid (article 74.2 of the International Code of Zoological Nomenclature^74^). Consequently, the name *Emydura victoriae* (Gray, 1842) is unambiguously validated for the red-faced *Emydura* species from the Victoria River, Northern Territory.

*Hydraspis australis* Gray, 1841 is another much debated taxon, currently treated as a junior synonym of *Em. macquarii* (Gray, 1830)^10,52^ or as a *nomen dubium*^9^. Previously, the name *Em. australis* has also been used for certain *Emydura* populations from northern Australia^14^. The differentiation of the mitogenome of the holotype of *H. australis* suggests that it is distinct from *E. macquarii* and may thus represent a valid species. Unfortunately, the type locality of *H. australis* is unknown^75^, even though it has been suggested to originate from “Western Australia”^10,52^ (which at the time included much of what we now call the Northern Territory) or the Macquarie River^76^. This situation requires wider sampling of fresh material to clarify the distribution of this species.

Besides these nomenclatural and taxonomic aspects, our sampling of mitogenomes of type specimens and fresh material allow some additional insights. Chelid turtles represent an iconic part of the extant fauna of South America and Australasia and are a prime example of a group of animals of Gondwana origin^77^. The topology of our phylogenies (Figs 1 and 2) corresponds to expectations from previous studies using less data or less taxa^26,78–80^. The mitogenomes of Australasian chelids support four ancient lineages, (1) *Pseudemydura umbrina*, (2) snake-necked and long-necked turtles of the genus *Chelodina*, (3) a clade comprised of the genera *Elseya*, *Myuchelys* (exclusive of *M. purvisi*) and *Emydura*, and (4) a clade containing *M. purvisi* and the monotypic genera *Elusor* and *Rheodytes*.

The mitogenome of *Pseudemydura umbrina* has it as the sister taxon to the remaining Australasian taxa, as recently revealed by a limited dataset of mitogenomes, including *P. umbrina*^26^. The mitogenomes of *Chelodina* species form a well-supported deep clade within the Australasian Chelidae. Within this genus, morphological and allozyme variation has been used to establish three major clades^10^, named as the subgenera (1) *Chelodina*, containing the long-necked species *C. canni*, *C. longicollis*, *C. mccordi*, *C. novaeguineae* (with *C. gunaleni* as junior synonym^10^), *C. pritchardi*, *C. reimanni*, and *C. steindachneri*; (2) *Macrochelodina*, corresponding to the snake-necked species *C. burrungandjii*, *C. expansa*, *C. parkeri*, and *C. rugosa* (the latter species thought to be synonymous with *C. kuchlingi* and *C. siebenrocki*^10^); and (3) the monotypic *Macrodiremys* for the snake-necked *C. colliei*.

Our mitogenome analysis conflicts with this arrangement in several respects. First, the mitochondrial genome of *C. parkeri* is sister to those of all remaining *Chelodina* species. This placement of *C. parkeri* is unexpected because it conflicts both with morphological groupings within *Chelodina*^10,51,55,56^ and allozyme evidence^57^, which both place *C. parkeri* in a clade also comprising *C. burrungandjii*, *C. expansa*, and *C. rugosa*^81^. Our mitogenomic phylogeny implies that the snake-necked condition is ancestral and retained by *C. burrungandjii*, *C. colliei*, *C. expansa*, *C. parkeri*, *C. rugosa*, and the newly recognized species *C. kuchlingi*, and secondarily reduced in the long-necked species *C. canni, C. longicollis*, the *C. novaeguineae* complex, and *C. steindachneri*. Alternatively, the snake-necked character state must have arisen at least three times independently (in *C. parkeri, C. colliei*, and the clade comprising *C. burrungandjii*, *C. expansa*, *C. kuchlingi*, and *C. rugosa*), leading to the phylogenetic interdigitation of the snake-necked and long-necked species, rendering the *Macrochelodina* subgenus polyphyletic (Figs 1 and 2).

Another unexpected finding refers to *C. expansa* and *C. longicollis*, two species highly distinct in morphology^14,55,58^ and allozyme profiles^57^. Two samples of each species yielded two highly distinct mitogenomes that occurred in two distinct phylogenetic positions. The mitogenome of the approximately 230-year-old holotype of *Testudo longicollis* is feebly differentiated from a *C. expansa* mitogenome from the Murray-Darling drainage (Chexp175). These two mitogenomes together are sister to a clade containing the mitogenomic sequences of several taxa from Australia and New Guinea, including several type specimens (*Chelodina* subgenus: *C. canni*, *C. gunaleni*, *C. m. mccordi*, *C. m. roteensis*, *C. m. timorensis*, *C. m. timorlestensis*, *C. novaeguineae*, *C. pritchardi*, *C. reimanni*; *Macrochelodina* subgenus: *C. burrungandjii*) and the virtually identical mitogenomes of a *C. expansa* (KY705230) from the Dawson River in Queensland and another previously published mitogenome sequence of *C. longicollis* from the Australian Capital Territory (KJ713173)^47^. This implies for *C. expansa* and *C. longicollis* multiple mitochondrial introgression events, a finding in line with the yet unpublished results of the PhD thesis of Hodges^82^. This situation also suggests that mitochondrial capture could have occurred in further *Chelodina* lineages. If this hypothesis is true, it could explain the polyphyly of the mitogenomes of the *Chelodina* subgenera. This is also supported by the observation of natural hybridization involving other *Chelodina* species (*C. burrungandjii* × *C. canni*, *C. burrungandjii* × *C. rugosa*, *C. canni* × *C. longicollis*, *C. canni* × *C. rugosa*)^10,15,57,83^, leading in the Arnhem Land region to widespread mitochondrial introgression of *C. burrungandjii* with *C. rugosa* haplotypes^83^.

Another deeply divergent clade identified by our analysis comprises the species of *Elseya*, *Emydura* and *Myuchelys* (excluding *Myuchelys purvisi*). The three major clades of *Elseya*, *Emydura* and *Myuchelys* are consistent with the findings of prior studies^71,84^. The Queensland east coast *Elseya albagula* is the sister taxon to the *Elseya* from northern Australia; within that northern clade *El. branderhorsti* and *El. flaviventralis* cluster together, a reflection of the close historical relationship between Australia and the island of New Guinea. Within the *Myuchelys* clade (excluding *M. purvisi*), *M. georgesi* is sister to a clade represented by *M. latisternum* east of the Great Dividing Range and *M. bellii* west of the range. Our data support two clades within *Emydura*. One northern clade contains *Emydura subglobosa* as sister taxon to a clade comprising the *Em. victoriae* (Emvic245) from the Daly River, Northern Territory, and *Em. tanybaraga*, which is consistent with earlier findings based on allozymes^71,84^. Another mitogenome, identified as “*Emydura subglobosa*”, was downloaded from GenBank (KC692462) and seems to originate from a trade turtle without locality data. It is virtually identical to our mitogenome of *Em. tanybaraga*. Since *Em. subglobosa* and *Em. tanybaraga* are morphologically similar, a misidentification seems likely. The second clade within *Emydura* includes the holotype of *Em. australis* and the holotype of *Em. victoriae* (previously misidentified as paralectotype) as one clade, and the southern *Em. macquarii*, represented by the mitogenomes of a fresh sample (Emmac119, Murray River, New South Wales) and the mitogenome of the historical museum specimen that was previously misidentified as the lectotype of *Hydraspis victoriae* as another clade (see discussion of species identification above).

The paraphyly of the genus *Myuchelys* supported by our data is consistent with the findings of past studies using mitochondrial data^85,86^. Using one nuclear and two mitochondrial markers, Le *et al*.^86^ concluded that *Myuchelys purvisi*, *Elusor macrurus* and *Rheodytes leukops* constitute the successive sister taxa of all other short-necked Australasian chelids (*Pseudemydura umbrina* was not studied), rendering *Myuchelys* polyphyletic. Based on this topology, Le *et al*.^86^ removed *M. purvisi* from *Myuchelys* and placed it in the newly described genus *Flaviemys*. Using one mitochondrial and 13 nuclear loci, Spinks *et al*.^87^ found conflicting evidence from the two marker systems in that they revealed for the mitochondrial COI gene the same topology that we did. However, based on the nuclear loci *M. purvisi* clustered with high support among the other studied *Myuchelys* species, and Spinks *et al*.^87^ therefore relegated *Flaviemys* Le *et al*.^86^ into the synonymy of *Myuchelys* Thomson & Georges, 2009.

We suggest that the conflicting topologies of the well supported nuclear and mitochondrial trees reflect ancient mitochondrial capture, and that *M. purvisi* carries the mitochondrial heritage of an extinct lineage related to *El. macrurus* and *R. leukops*. If this hypothesis is correct, *M. purvisi* could represent the oldest case of mitochondrial capture. Fossils identifiable as *Myuchelys* date back to the Albian (Lower Cretaceous, 113–100.5 Ma)^88^, and fossils representing the lineage of *M. purvisi* date back to the upper Eocene or Oligocene (41–27 Ma)^89^, implying their long distinctness from the *Elusor* and *Rheodytes* lineages. Mitochondrial introgression, resulting in cytonuclear discordance, is widespread in animals^90–96^ and also known for a number of turtle species (Geoemydidae: *Cyclemys*, *Malayemys*^97–99^; Emydidae: *Actinemys*, *Emys*, *Emydoidea*, *Graptemys*^100–102^), among them is also the *Chelodina* genus (Chelidae; see above). However, mitochondrial capture, i.e. the complete replacement of the original mitochondrial genome with that of another species through introgressive hybridization, is much rarer. Cases have been described for insects^103^, freshwater fishes^104–106^, newts^107^, frogs^108–111^, turtles^97,100–102^, lizards^112^, snakes^113^, and bovids^114–117^, with the oldest case (12 Ma) referring to emydid turtles^100^.

In summary, we have resolved a number of important issues that have confused the taxonomy of this family in Australasia. We were able to assign the holotype of *Phrynops bellii* to populations of *Myuchelys* from the rivers of the Murray-Darling Basin draining west from Australia’s Great Dividing Range, confirming is previous identification based on circumstantial evidence inferred from the travels of the person who collected it^14^. We clarified the confusion over the name-bearing type of *Emydura victoriae* (Gray, 1842), and recognized the specimen previously misidentified as its paralectotype as the holotype. The deep divergence between the mitogenome of the holotype from a mitogenome of a fresh sample of *Em. victoriae* from the Daly River, Northern Territory, suggests together with differences in allozyme profiles and coloration^71^ that two distinct species are currently lumped together under the name *Em. victoriae*. The holotype of *Emydura australis* (Gray, 1841) remains of uncertain geographic provenance but is considered to represent a valid species. *Chelodina kuchlingi* Cann, 1997, which was described from a single specimen of uncertain provenance, is confirmed as distinct based on its mitogenome, and the recent assignment of the holotype of *Chelodina oblonga* Gray, 1841 to northern Australian populations is brought into question, requiring the resurrection of *C. rugosa* Ogilby, 1890 for those populations. However, the genetic divergences within the *C. rugosa* complex suggest the existence of multiple species. We identified only two taxa within *C. mccordi* Rhodin, 1994, at the level of subspecies, from the islands of Roti and Timor, respectively.

We have combined the mitogenomes of the studied types with those of fresh material to improve our understanding of the phylogenetic relationships of this morphologically conservative family of turtles, providing evidence for multiple old mitochondrial introgressions in chelid turtles. This study established the value of generating diagnostic sequences from type specimens of taxa with a confused and uncertain taxonomic and nomenclatural history. DNA sequencing of name-bearing type specimens is the gold standard for assigning extant populations to a named entity^7^ and enables the setting of clear and essential distinctions between newly discovered forms and those that have already been described.

### ZooBank Registration

This published work and the nomenclatural acts it contains have been registered in ZooBank, the online registration system for the International Commission on Zoological Nomenclature (ICZN). The ZooBank LSID (Life Science Identifier) can be resolved and the associated information can be viewed through any standard web browser by appending the LSID to the prefix http://zoobank.org. The LSID for this publication is as follows: urn:lsid:zoobank.org:pub: http://zoobank.org/References/2C8F580D-527B-44A2-BF2C-1BC9CD02D9E1.

## Supplementary information

Supplementary Information

## Data Availability

All data analyzed during this study are included in this published article and its Supplementary Information files. DNA sequences have been uploaded to the European Nucleotide Archive (ENA) and are available under the accession numbers listed in Supplementary Tables S1 and S2.

## References

[1] Ceballos G (2015). Accelerated modern human–induced species losses: Entering the sixth mass extinction. Sci. Adv..

[2] Darlington PJ (1971). The carabid beetles of New Guinea. Part IV. General consideration; analysis and history of fauna; taxonomic supplement. Bull. Mus. Comp. Zool..

[3] Hofreiter M (2014). The future of ancient DNA: technical advances and conceptual shifts. BioEssays.

[4] Grealy A, Rawlence NJ, Bunce M (2017). Time to spread your wings: a review of the avian ancient DNA field. Genes.

[5] Woods R, Marr MM, Brace S, Barnes I (2017). The small and the dead: a review of ancient DNA studies analysing micromammal species. Genes.

[6] Estrada O, Breen J, Richards SM, Cooper A (2018). Ancient plant DNA in the genomic era. *Nature*. Plants.

[7] Renner SS (2016). A return to Linnaeus’s focus on diagnosis, not description: the use of DNA characters in the formal naming of species. Syst. Biol..

[8] Mittermeier, R. A., Robles Gil, P. & Mittermeier, C. G. (eds) *Megadiversity—Earth’s Biologically Wealthiest Nations* (CEMEX/Agrupación Sierra Madre, 1997).

[9] TTWG [Turtle Taxonomic Working Group]. *Turtles of the World. Annotated Checklist and Atlas of Taxonomy, Synonymy, Distribution, and Conservation Status. Eighth Edition* (Chelonian Research Foundation and Turtle Conservancy, Chelon. Res. Monogr. 7, 2017).

[10] Georges A, Thomson S (2010). Diversity of Australasian freshwater turtles, with an annotated synonymy and keys to species. Zootaxa.

[11] Cann J, Legler JM (1994). The Mary River tortoise: a new genus and species of short-necked chelid from Queensland, Australia (Testudines; Pleurodira). Chelon. Conserv. Biol..

[12] Rhodin AGJ (1994). Chelid turtles of the Australasian Archipelago: I. A new species of *Chelodina* from southeastern Papua New Guinea. Breviora.

[13] Rhodin AGJ (1994). Chelid turtles of the Australasian Archipelago: II. A new species of *Chelodina* from Roti Island, Indonesia. Breviora.

[14] Cann, J. *Australian Freshwater Turtles* (Beaumont Publishing, 1998).

[15] McCord WP, Thomson SA (2002). A new species of *Chelodina* (Testudines: Pleurodira: Chelidae) from northern Australia. J. Herpetol..

[16] Thomson S, Kennett R, Georges A (2000). A new long-necked turtle (Testudines: Chelidae) from the Arnhem Land Plateau, Northern Territory, Australia. Chelon. Conserv. Biol..

[17] Thomson S, Georges A, Limpus CJ (2006). A new freshwater turtle in the genus *Elseya* (Testudines: Chelidae) from central coastal Queensland, Australia. Chelon. Conserv. Biol..

[18] Thomson S, Amepou Y, Anamiato J, Georges A (2015). A new species and subgenus of *Elseya* (Testudines: Pleurodira: Chelidae) from New Guinea. Zootaxa.

[19] Georges, A. & Thomson, S. Evolution and zoogeography of the Australian freshwater turtles in *Evolution and Zoogeography of* Australasian *Vertebrates* (eds Merrick, J. R., Archer, M., Hickey, G. & Lee, M.), 291–308 (Australian Scientific Publishing, 2006).

[20] Thomson S, Georges A (2009). *Myuchelys* gen. nov. – a new genus for *Elseya latisternum* and related forms of Australian freshwater turtle. Zootaxa.

[21] Thomson S, Georges A (2016). A new species of freshwater turtle of the genus *Elseya* (Testudinata: Pleurodira: Chelidae) from the Northern Territory of Australia. Zootaxa.

[22] Rhodin AGJ (2015). Comment on *Spracklandus* Hoser, 2009 (Reptilia, Serpentes, Elapidae): request for confirmation of the availability of the generic name and for the nomenclatural validation of the journal in which it was published. Bull. Zool. Nomencl..

[23] Thomson SA (2018). Taxonomy based on science is necessary for global conservation. PLoS Biol..

[24] Thomson S (2000). The identification of the holotype of *Chelodina oblonga* (Testudinata: Chelidae) with a discussion of taxonomic implications. Chelon. Conserv. Biol..

[25] Shaw, G. *Zoology of New Holland, Vol. I* (J. Davis, 1794).

[26] Zhang X, Unmack PJ, Kuchling G, Wang Y, Georges A (2017). Resolution of the enigmatic phylogenetic relationship of the critically endangered western swamp tortoise *Pseudemydura umbrina* (Pleurodira: Chelidae) using a complete mitochondrial genome. Mol. Phylogenet. Evol..

[27] Meyer M, Kircher M (2010). Illumina sequencing library preparation for highly multiplexed target capture and sequencing. Cold Spring Harb. Protoc..

[28] Fortes, G. G. & Paijmans, J. L. A. Analysis of whole mitogenomes from ancient samples in *Whole Genome Amplification. Methods in Molecular Biology, 1347* (ed Kroneis, T.), 179–195 (Humana Press, 2015). 10.1007/978-1-4939-2990-0_13 26374318

[29] Maricic T, Whitten M, Pääbo S (2010). Multiplexed DNA sequence capture of mitochondrial genomes using PCR products. PLoS ONE.

[30] Horn, S. Target enrichment via DNA hybridization capture in *Ancient DNA: Methods and Protocols. Methods in Molecular Biology, 840* (eds Shapiro, B. & Hofreiter, M.), 177–188 (Springer, 2012). 10.1007/978-1-61779-516-9_21 22237535

[31] Fulton, T. L. Setting up an ancient DNA laboratory in *Ancient DNA: Methods and Protocols. Methods in Molecular Biology, 840* (eds Shapiro, B. & Hofreiter, M.), 1–11 (Springer, 2012). 10.1007/978-1-61779-516-9_1 22237515

[32] Jiang H, Lei R, Ding SW, Zhu S (2014). SKEWER: a fast and accurate adapter trimmer for next-generation sequencing paired-end reads. BMC Bioinformatics.

[33] Bushnell B, Rood J, Singer E (2017). BBMERGE – accurate paired shotgun read merging via overlap. PLoS ONE.

[34] Hahn C, Bachmann L, Chevreux B (2013). Reconstructing mitochondrial genomes directly from genomic next-generation sequencing reads—a baiting and iterative mapping approach. Nucl. Acids Res..

[35] Chevreux B, Wetter T, Suhai S (1999). Genome sequence assembly using trace signals and additional sequence information in *Computer Science and Biology*. Proceedings of the German Conference on Bioinformatics (GCB).

[36] Milne I (2013). Using TABLET for visual exploration of second-generation sequencing data. Brief. Bioinform..

[37] Bernt M (2013). MITOS: improved de novo metazoan mitochondrial genome annotation. Mol. Phylogenet. Evol..

[38] Hall TA (1999). BioEdit: a user-friendly biological sequence alignment editor and analysis program for Windows 95/98/NT. Nucl. Acids Symp. Ser..

[39] Thompson JD, Higgins DG, Gibson TJ (1994). CLUSTAL W: improving the sensitivity of progressive multiple sequence alignment through sequence weighting, position specific gap penalties and weight matrix choice. Nucl. Acids Res..

[40] Stamatakis A (2014). RAxML version 8: a tool for phylogenetic analysis and post-analysis of large phylogenies. Bioinformatics.

[41] Ronquist F (2011). MRBAYES 3.2: efficient Bayesian phylogenetic inference and model choice across a large model space. Syst. Biol..

[42] Lanfear R, Frandsen PB, Wright AM, Senfeld T, Calcott B (2016). PARTITIONFINDER 2: new methods for selecting partitioned models of evolution for molecular and morphological phylogenetic analyses. Mol. Biol. Evol..

[43] Rambaut A, Drummond AJ, Xie D, Baele G, Suchard MA (2018). Posterior summarisation in Bayesian phylogenetics using TRACER 1.7. Syst. Biol..

[44] Kumar S, Stecher G, Tamura K (2016). MEGA7: Molecular Evolutionary Genetics Analysis version 7.0 for bigger datasets. Mol. Biol. Evol..

[45] Fritz U (2002). Herpetology and herpetological type specimens at the Museum für Tierkunde Dresden with a bibliography of herpetological contributions by Fritz Jürgen Obst. Faun. Abh..

[46] Pereira SL (2000). Mitochondrial genome organization and vertebrate phylogenetics. Genet. Mol. Biol..

[47] Zhang X, Georges A (2014). A complete mitochondrial genome sequence for the Australian turtle, *Chelodina longicollis*, obtained using 454-pyrosequencing. Conserv. Genet. Resour..

[48] Thomson SA (2006). Case 3351. *Chelodina rugosa* Ogilby, 1890 (currently *Macrochelodina rugosa*; Reptilia, Testudines): proposed precedence over *Chelodina oblonga* Gray, 1841. Bull. Zool. Nomencl..

[49] Wermuth, H. & Mertens, R. *Schildkröten, Krokodile, Brückenechsen* (VEB Gustav Fischer, 1961).

[50] Wermuth H, Mertens R (1977). Testudines, Crocodylia, Rhynchocephalia. Tierreich.

[51] Burbidge AA, Kirsch JAW, Main AR (1974). Relationships within the Chelidae (Testudines: Pleurodira) of Australia and New Guinea. Copeia.

[52] Cogger, H., Cameron, E., Sadlier, R. & Eggler, P. *The Action Plan for Australian Reptiles* (Environment Australia, 1983).

[53] Siebenrock F (1909). Synopsis der rezenten Schildkröten unter Berücksichtigung der in historischer Zeit ausgestorbenen Arten. Zool. Jahrb..

[54] Siebenrock F (1914). Eine neue *Chelodina*-Art aus Westaustralien. Anz. Akad. Wiss. Wien (Math.-Naturw. Kl.).

[55] Goode, J. *Freshwater Tortoises of Australia and New Guinea (in the Family Chelidae)* (Lansdowne Press, 1967).

[56] Rhodin AGJ, Mittermeier RA (1976). *Chelodina parkeri*, a new species of chelid turtle from New Guinea, with a discussion of *Chelodina siebenrocki* Werner, 1901. Bull. Mus. Comp. Zool..

[57] Georges A, Adams M, McCord W (2002). Electrophoretic delineation of species boundaries within the genus *Chelodina* (Testudines: Chelidae) of Australia, New Guinea and Indonesia. Zool. J. Linn. Soc..

[58] Cann, J. & Sadlier, R. *Freshwater Turtles of Australia* (EOC Wear & Publishing and CSIRO, 2017).

[59] TTWG [Turtle Taxonomic Working Group] (2014). Turtles of the world, 7th edition: annotated checklist of taxonomy, synonymy, distribution with maps, and conservation status. Chelon. Res. Monogr..

[60] Cann J (1997). Kuchling’s long-neck Turtle. Monitor.

[61] Iverson JB, Ernst CH, Gotte S, Lovich JE (1989). The validity of *Chinemys megalocephala* (Testudines: Batagurinae). Copeia.

[62] Fritz U (1992). Zur innerartlichen Variabilität von *Emys orbicularis* (Linnaeus, 1758). 2. Variabilität in Osteuropa und Redefinition von *Emys orbicularis orbicularis* (Linnaeus, 1758) und *E. o. hellenica* (Valenciennes, 1832). Zool. Abh..

[63] Ernst, C. H. & Lovich, J. E. *Turtles of the United States and Canada* (Johns Hopkins University Press, 2009).

[64] Ennen JR, Lovich JE, Kreiser BR, Selman W, Qualls CP (2010). Genetic and morphological variation between populations of the Pascagoula map turtle (*Graptemys gibbonsi*) in the Pearl and Pascagoula Rivers with description of a new species. Chelon. Conserv. Biol..

[65] Dalrymple GH (1977). Intraspecific variation in the cranial feeding mechanism of turtles of the genus *Trionyx* (Reptilia, Testudines, Trionychidae). J. Herpetol..

[66] Rhodin, A. G. J. & Genorupa, V. R. Conservation status of freshwater turtles in Papua New Guinea in *Asian Turtle Trade: Proceedings of a Workshop on Conservation and Trade of Freshwater Turtles and Tortoises in Asia* (eds van Dijk, P. P., Stuart, B. L. & Rhodin, A. G. J.), 129–136 (Chelonian Research Foundation, Chelon. Res. Monogr. 2, 2000).

[67] McCord WP, Joseph-Ouni M (2007). A new species of *Chelodina* (Testudines: Chelidae) from southwestern New Guinea (Papua, Indonesia). Reptilia (GB).

[68] Iverson JB, Thomson SA, Georges A (2001). The validity of the taxonomic changes for turtles proposed by Wells and Wellington. J. Herpetol..

[69] Wells RW, Wellington CR (1985). A classification of the Amphibia and Reptilia of Australia. Austral. J. Herpetol., Suppl. Ser..

[70] Gray JE (1842). Description of some hitherto unrecorded species of Australian reptiles and batrachians. Zool. Misc..

[71] Georges A, Adams M (1996). Electrophoretic delineation of species boundaries within the short-necked chelid turtles of Australia. Zool. J. Linn. Soc..

[72] Boulenger, G. A. *Catalogue of the Chelonians, Rhynchocephalians, and Crocodiles in the British Museum (Natural History)* (Trustees of the British Museum, 1889).

[73] Gray, J. E. *Catalogue of the Tortoises, Crocodiles, and Amphisbaenians in the Collection of the British Museum* (Trustees of the British Museum, 1844).

[74] ICZN [International Commission on Zoological Nomenclature]. *International Code of Zoological Nomenclature. Fourth Edition* (International Trust for Zoological Nomenclature, 1999).

[75] Gray, J. E. A catalogue of the species of reptiles and amphibia hitherto described as inhabiting Australia, with a description of some new species from Western Australia, and some remarks on their geographical distribution. In *Journals of Two Expeditions of Discovery in Northwest and Western Australia, Vol. 2, Appendix E* (ed Grey, G.), 422–449 (T. and W. Boone, 1841).

[76] Gray JE (1872). On the genus *Chelymys* and its allies from Australia. Proc. Zool. Soc. London.

[77] de la Fuente, M. S., Sterli, J. & Maniel, I. *Origin, Evolution and Biogeographic History of South American Turtles* (Springer, Springer Earth System Sciences, 2014).

[78] Georges A, Birrel J, Saint KM, McCord W, Donnellan SC (1999). A phylogeny for side-necked turtles (Chelonia: Pleurodira) based on mitochondrial and nuclear gene sequence variation. Biol. J. Linn. Soc..

[79] Pereira AG, Sterli J, Moreira FRR, Schargo CG (2017). Multilocus phylogeny and statistical biogeography clarify the evolutionary history of major lineages of turtles. Mol. Phylogenet. Evol..

[80] Shaffer HB, McCartney-Melstad E, Near TJ, Mount GG, Spinks PQ (2017). Phylogenomic analyses of 539 highly informative loci dates a fully resolved time tree for the major clades of living turtles (Testudines). Mol. Phylogenet. Evol..

[81] Thomson S (2011). *Chelodina burrungandjii* Thomson, Kennett, and Georges 2000 – Sandstone snake-necked turtle. Chelon. Res. Monogr..

[82] Hodges, K. M. *Recent evolutionary history of the Australian freshwater turtles Chelodina expansa and Chelodina longicollis* (PhD Thesis, University of Adelaide, 2015).

[83] Alacs, A. A. *Forensics, Phylogeography and Population Genetics: A Case Study using the Northern Snake-necked turtle, Chelodina rugosa* (PhD Thesis, University of Canberra, 2008).

[84] Georges A, Adams M (1992). A phylogeny for Australian chelid turtles based on allozyme electrophoresis. Austral. J. Zool..

[85] Fielder, D., Vernes, K., Alacs, E. & Georges, A. Mitochondrial variation among Australian freshwater turtles (genus *Myuchelys*), with special reference to the endangered *M. bellii*. *Endanger. Species Res*. **17**, 63–71 (2012).

[86] Le M (2013). Resolving the phylogenetic history of the short-necked turtles, genera *Elseya* and *Myuchelys* (Testudines: Chelidae) from Australia and New Guinea. Mol. Phylogenet. Evol..

[87] Spinks PQ, Georges A, Shaffer HB (2015). Phylogenetic uncertainty and taxonomic re-revisions: an example from the Australian short-necked turtles (Testudines: Chelidae). Copeia.

[88] Smith ET (2010). Early Cretaceous chelids from Lightning Ridge, New South Wales. Alcheringa.

[89] Warren JW (1969). Chelid turtles from the mid-Tertiary of Tasmania. J. Paleontol..

[90] Funk DJ, Omland KE (2003). Species-level paraphyly and polyphyly: Frequency, causes, and consequences, with insights from Animal Mitochondrial DNA. Annu. Rev. Ecol. Evol..

[91] Ballard JWO, Whitlock MC (2004). The incomplete natural history of mitochondria. Mol. Ecol..

[92] Chan KMA, Levin SA (2005). Leaky prezygotic isolation and porous genomes: rapid introgression of maternally inheritance DNA. Evolution.

[93] Currat M, Ruedi M, Petit RJ, Excoffier L (2008). The hidden side of invasions: massive introgression by local genes. Evolution.

[94] Zink RM, Barrowclough GF (2008). Mitochondrial DNA under siege in avian phylogeography. Mol. Ecol..

[95] Toews DPL, Brelsford A (2012). The biogeography of mitochondrial and nuclear discordance in animals. Mol. Ecol..

[96] Sloan DB, Havird JC, Sharbrough J (2017). The on-again, off-again relationship between mitochondrial genomes and species boundaries. Mol. Ecol..

[97] Fritz U (2008). Diversity of the Southeast Asian leaf turtle genus *Cyclemys*: how many leaves on its tree of life?. Zool. Scr..

[98] Ihlow F (2016). Integrative taxonomy of Southeast Asian snail-eating turtles (Geoemydidae: *Malayemys*) reveals a new species and mitochondrial introgression. PLoS ONE.

[99] Vamberger M (2017). The leaf turtle population of Phnom Kulen National Park (northwestern Cambodia) has genetic and morphological signatures of hybridization. J. Zool. Syst. Evol. Res..

[100] Spinks PQ, Shaffer HB (2009). Conflicting mitochondrial and nuclear phylogenies for the widely disjunct *Emys* (Testudines: Emydidae) species complex, and what they tell us about biogeography and hybridization. Syst. Biol..

[101] Praschag P, Ihlow F, Flecks M, Vamberger M, Fritz U (2017). Diversity of North American map and sawback turtles (Testudines: Emydidae: *Graptemys*). Zool. Scr..

[102] Thomson, R. C., Spinks, P. Q. & Shaffer, H. B. Molecular phylogeny and divergence of the map turtles (Emydidae: *Graptemys*). *Mol. Phylogenet. Evol*. **121**, 61–70 (2018). 10.1016/j.ympev.2017.11.012 29242165

[103] Linnen CR, Farrell BD (2007). Mitonuclear discordance is caused by rampant mitochondrial introgression in *Neodiprion* (Hymenoptera: Diprionidae) sawflies. Evolution.

[104] Nevado B (2009). Complete mitochondrial DNA replacement in a Lake Tanganyika cichlid fish. Mol. Ecol..

[105] Tang Q-Y, Liu S-Q, Yu D, Liu H-Z, Danley PD (2012). Mitochondrial capture and incomplete lineage sorting in the diversification of balitorine loaches (Cypriniformes, Balitoridae) revealed by mitochondrial and nuclear genes. Zool. Scr..

[106] Perea S, Vukić J, Šanda R, Doadrio I (2016). Ancient mitochondrial capture as factor promoting mitonuclear discordance in freshwater fishes: a case study in the genus *Squalius* (Actinopterygii, Cyprinidae) in Greece. PLoS ONE.

[107] Babik W (2005). Phylogeography of two European newt species – discordance between mtDNA and morphology. Mol. Ecol..

[108] Bryson RW, Nieto-Montes de Oca A, Jaeger JR, Riddle BR (2010). Elucidation of cryptic diversity in a widespread Neartic treefrog reveals episodes of mitochondrial gene capture as frogs diversified across a dynamic landscape. Evolution.

[109] Bryson RW, Smith BT, Nieto-Montes de Oca A, García-Vázquez UO, Riddle BR (2014). The role of mitochondrial introgression in illuminating the evolutionary history of Neartic treefrogs. Zool. J. Linn. Soc..

[110] Komaki S (2015). Robust molecular phylogeny and palaeodistribution modelling resolve a complex evolutionary history: glacial cycling drove recurrent mtDNA introgression among *Pelophylax* frogs in East Asia. J. Biogeogr..

[111] Fouquet, A. *et al*. Phenotypic and life history diversification in Amazonian frogs despite past introgressions. *Mol. Phylogenet. Evol*. **130**, 169–180 (2019). 10.1016/j.ympev.2018.09.010 30292694

[112] Leaché AD, McGuire JA (2006). Phylogenetic relationships of horned lizards (*Phrynosoma*) based on nuclear and mitochondrial data: evidence for a misleading mitochondrial gene tree. Mol. Phylogenet. Evol..

[113] Barbanera F (2009). Molecular phylogeography of the asp viper *Vipera aspis* (Linnaeus, 1758) in Italy: evidence for introgressive hybridization and mitochondrial DNA capture. Mol. Phylogenet. Evol..

[114] Ropiquet A, Hassanin A (2006). Hybrid origin of the Pliocene ancestor of wild goats. Mol. Phylogenet. Evol..

[115] Hassanin A, Ropiquet A (2007). Resolving a zoological mystery: the kouprey is a real species. Proc. R. Soc. B.

[116] Hassanin A, An J, Ropiquet A, Nguyen TT, Couloux A (2013). Combining multiple autosomal introns for studying shallow phylogeny and taxonomy of Laurasiatherian mammals: application to the tribe Bovini (Cetartiodactyla, Bovidae). Mol. Phylogenet. Evol..

[117] Hassanin A (2018). Multi-locus phylogeny of the tribe Tragelaphini (Mammalia, Bovidae) and species delimitation in bushbuck: evidence for chromosomal speciation mediated by interspecific hybridization. Mol. Phylogenet. Evol..

