# Supplementary material for: Mitogenomics of historical type specimens of Australasian turtles: clarification of taxonomic confusion and old mitochondrial introgression
Source: Sci Rep. 2019 Apr 9;9:5841. doi: 10.1038/s41598-019-42310-x (PMC6456567; doi:10.1038/s41598-019-42310-x)
Supplement: Supplementary file 1 — Supplementary Information [file 41598_2019_42310_MOESM1_ESM.pdf]

Mitogenomics of historical type specimens of Australasian turtles: clarification of taxonomic confusion and old mitochondrial introgression

Christian Kehlmaier, Xiuwen Zhang, Arthur Georges, Patrick D. Campbell, Scott Thomson, Uwe Fritz

Scientific Reports

https://doi.org/10.1038/s41598-019-42310-x

Supplementary Information

Methods for historical material

All historical samples were either ethanol- or dry-preserved tissues. For weighing ethanol-preserved tissues, samples were transferred into 1.5 ml vials and dried in a thermomixer set to 56°C for some minutes. DNA was extracted with the Qiagen DNeasy Blood & Tissue Kit (Qiagen, Hilden, Germany) using the 'Purification of Total DNA from Animal Tissues (Spin-Column) Protocol' with the following modifications: The amount of buffer ATL (step 1), buffer AL (step 3) and ethanol (step 3) was doubled, and 30 µl proteinase K was used for lysis, which lasted 18 hr in a thermomixer set to 56°C and 450 rpm. All samples were eluted in two times 50 µl buffer AE and 10 min incubated at room temperature. DNA concentration was measured with a Qubit 3.0 Fluorometer (Thermo Fisher Scientific, Waltham, MA) using Qubit HS (High Sensitivity) assays. The length of the DNA molecules was determined with the Agilent 4200 TapeStation System (Agilent, Santa Clara, CA), using the genomic screen tape assay. The amount of tissue used for extraction ranged from 3.8 mg to 35.1 mg (Table S6).

Fresh samples and the tissues used for bait library preparation were buffer- (10% EDTA, 0.5% NaF, 0.5% Thymol, 1% Tris, pH=7.5) or ethanol-preserved (96%). Buffer-preserved samples were washed twice with 300 µl 1x TE by centrifugation for 2 min at 13,000 rpm prior to lysis. Ethanol-preserved tissue was transferred into 1.5 ml vials and dried in a thermomixer set to 50°C for some minutes. DNA was extracted with the Analytik Jena innuPREP DNA Mini Kit (Analytik Jena, Jena, Germany: Protocol 1), adding an RNAse digestion after lysis with 10 µl RNAse (10 mg/µl) for 30 min at 37°C. All samples were eluted twice using 50 µl elution buffer pre-warmed to 70°C with 10 min incubation at room temperature. DNA concentration was measured with HS assays. Fragment length distribution was analysed with the TapeStation.

NGS library preparation followed Meyer & Kircher[1] with modifications by Fortes & Paijmans[2]. Due to the highly fragmented DNA, no enzymatic or ultrasonic fragmentation was performed. The protocol allows a starting quantity between 100 pg and 1 µg of double-stranded DNA and has a conversion rate of DNA molecules into NGS library molecules of 10–20%[1]. Each NGS library was tagged with a unique eight nucleotide long barcode sequence incorporated into the P7-adapter of the library molecule (index source: https://bioinf.eva.mpg.de/multiplex/index_8nt_dist5.txt) and amplified using four units of Herculase II Fusion DNA Polymerase (Agilent) in a single 80 µl PCR reaction. After reaction clean-up, using the MinElute PCR Purification Kit (Qiagen) with an elution in two times 10 µl buffer EB with a 10 min incubation time at room temperature, the indexed NGS libraries were analyzed for their concentration (HS assays) and fragment length distribution, the latter with the 4200 TapeStation System (Agilent) using the D1000 screen tape assay (D1000).

Bait libraries were prepared according to Maricic *et al*.[3] and Horn[4]. This included long-range PCR amplification of high molecular weight DNA from one sample of *Chelodina longicollis* (Museum of Zoology Dresden, sample MTD 7251), *Elseya novaeguineae* (MTD 137) and *Emydura australis* (MTD 3933) each, and subsequent ultrasonication of the PCR products to approximately 150 bp using a Covaris M220 ultrasonicator (Covaris, Woburn, USA). Long-range PCR primers (Table S7) were designed using OLIGO EXPLORER 1.1.2 based on a consensus sequence inferred from an alignment of multiple modern mt-genomes of each genus plus GenBank accession number HQ172157. Two sets of overlapping primer pairs (overlap: 106 bp) covered slightly more than 16 kbp of the mitochondrial genome ranging from the end of the phenylanaline-tRNA to the middle of the control region. A third primer pair failed to amplify a region covering the second half of the control region and part of 12S, probably due to the presence of large repetitive DNA blocks in the control region. Long-range PCR reactions were performed in 50 µl volumes, containing 5–10 ng of DNA and 1 unit of TaKaRa LA Taq DNA Polymerase, Hot-Start Version (Clontech Laboratories Inc., Mountain View, CA), following the reaction mixture recommended by the manufacturer. PCR conditions comprised an initial denaturation at 93°C for 3 min, followed by 35–40 cycles of 93°C for 15 sec, an optimized annealing temperature for 0:30, 68°C for 10 min, before a final elongation at 68°C for 20 min (Table S8). PCR products were visualized and, if necessary, excised from a 2% agarose gel and purified using the peqGOLD Gel Extraction Kit (PEQLAB Biotechnologie GmbH, Erlangen, Germany). To verify the authenticity of the long-range PCR products, the amplified fragments were Sanger-sequenced prior to bait library preparation, using well established sequencing primers for the 12S and cyt *b* regions (Table S7) and standard laboratory procedures[5]. For cycle sequencing, the total reaction volume of 10 µl contained 2 µl sequencing buffer, 1 µl premix, 0.5 µM of the respective primer, 1 µl DNA template, and ultrapure H_2O. Using the ABI PRISM Big Dye Terminator v.3.1 Cycle Sequencing Kit (Thermo Fisher Scientific), 25 cycles were performed at 96°C for 10 min, 50°C for 5 sec and 60°C for 4 min. Reaction products were purified by gel filtration using the Performa DTR V3 96-Well Short Plate Kit (EdgeBio, Gaithersburg, MD) and 400 µl of a 5% Sephadex solution (GE Healthcare, Munich, Germany). Sequencing was performed on an ABI 3730 Genetic Analyser (Thermo Fisher Scientific).

To enrich sample libraries for mitochondrial DNA fragments, two-rounds of in-solution hybridization capture were performed according to Maricic *et al*.[3] and Horn[4], using the previously produced bait libraries. The method was designed for an amount of DNA library ranging from 100 ng to 1 µg and includes a library amplification step after each round using Herculase II Fusion DNA Polymerase (Agilent).

All 23 captured DNA libraries (including extraction and library blanks) were sequenced in the Molecular Laboratory of Senckenberg Dresden in a single run on an Illumina MiSeq sequencing platform using a 2x 300 cycle sequencing kit. The individual NGS libraries were pooled so that the number of aimed reads per library ranged from 1 million to 1.25 million.

After assembling the mitochondrial genomes and aligning them with the GenBank data (see main text for details), the annotation of the final assembly resulted in the following amendments: (1) stop codons of coding genes were excluded from the coding regions as these do not code for any amino acid; (2) gene overlap between coding regions were deleted from the alignment as these short regions cannot be attributed to a single gene, underlie a distinct evolutionary model, and, most importantly, contained a frame shift in one instance that made a sensible alignment impossible. This was the case between atp8 and atp6, atp6 and coxIII, as well as for ND4L and ND4. (3) Alignment positions that caused frame shifts in coding regions were removed from the alignment, i.e., twice in ND3 and twice in ND4L. (4) An unalignable 141-bp-long non-coding stretch between ND5 and ND6 was excluded. (5) MITOS accounted for a split/duplicated coxI gene in sample Chste238 (cox1–0 + strand 1521 bp; cox1–1 – strand 51 bp). The 51 bp on the negative strand were not taken into account here. In total, 184 sites were excluded (Table S9) and the final alignment was reduced from 16,473 bp to 16,289 bp (alignment 'FULL'). Finally, all coding regions were checked for the presence of internal stop codons with MEGA7[6], and a second alignment was created that only includes the 13 coding genes of the mitochondrion and comprised 11,328 bp (alignment 'CDS'). Both alignments can be requested from the corresponding author. For information on sequencing reads and genome mapping of the processed type specimens, see Table S10.

Table S1. Studied chelid type specimens and European Nucleotide Archive (ENA) accession numbers of their mitogenomes. Museum acronyms: AMNH—American Museum of Natural History, New York; BMNH—The Natural History Museum, London; MSNG—Museo Civico di Storia Naturale di Genova; MTD—Museum für Tierkunde (Museum of Zoology), Senckenberg Natural History Collections Dresden, Dresden; MCZ—Museum of Comparative Zoology, Cambridge, Mass.; OUM—Oxford University Museum, Oxford, UK; WAM—Western Australian Museum, Perth; ZMB—Museum für Naturkunde, Berlin.

Nominal taxon	Original type locality	Type material	Condition	Preserved since	Accession number
Chelodina gunaleni McCord & Joseph-Ouni, 2007	Uta River Basin, Mimika District, Central Papua Province (Irian Jaya), Indonesia[7]	AMNH R160133, holotype	Alcohol-preserved	2007	LR215671
Chelodina kuchlingi Cann, 1997	Kalumburu, N. W. Australia (14°18'S 126°28'E)[8]	WAM R29411, holotype	Alcohol-preserved	*ca.* 1970	LR215672
Chelodina mccordi Rhodin, 1994	Danau Naloek, near Busalangga, *ca.* 11 km northeast of Tudameda and *ca.* 8 km southwest of Ba'a, elevation *ca.* 115 m, southwestern Roti Island (10°48' S 123°00' E), East Nusa Tenggara Province, Indonesia[9]	MCZ 176731, paratype	Alcohol-preserved	1994	LR215673
Chelodina mccordi roteensis McCord, Joseph-Ouni & Hagen, 2007	Lake Enduy, eastern Rote Island, East Nusa Tenggara Province, Indonesia[10]	AMNH R160132, holotype	Alcohol-preserved	2007	LR215674
Chelodina mccordi timorlestensis Kuchling, Rhodin, Ibarrondo & Trainor, 2007	Plain of Lake Iralalaro (= Lagoa Ira Lalaro) (*ca.* 08º28' S 127º07' E, *ca.* 334 m), east of Lospalos, Lautém District, Timor-Leste[11]	WAM 165888, holotype	Alcohol-preserved (tissues preserved prior to conservation)	2007	LR215675
Chelodina novaeguineae Boulenger, 1888	Katow, S. E. New Guinea[12]	BMNH 1946.1.22.36, lectotype (designated by Wells & Welling-ton)[13]	Dry, mounted	*ca.* 1885	LR215676
Chelodina oblonga Gray, 1841	Western Australia[14]	BMNH 1947.3.5.89, holotype	Dry, mounted	*ca.* 1840	LR215677
Chelodina reimanni Philippen & Großmann, 1990	Merauke River, West-Irian, New Guinea[15]	MTD 29178, holotype	Alcohol-preserved	1990	LR215678
Chelodina reimanni Philippen & Großmann, 1990	Merauke River, West-Irian, New Guinea[15]	MTD 42828, paratype	Alcohol-preserved	1990	LR215679
Chelodina siebenrocki Werner, 1901	Deutsch Neu-Guinea[16]	ZMB 16491, holotype	Alcohol-preserved	*ca.* 1900	LR215680

Table S1. *Continued*.

**Nominal taxon**	**Original type locality**	**Type material**	**Condition**	**Preserved since**	**Accession number**
Chelodina timorensis McCord, Joseph-Ouni & Hagen, 2007	Lake Ira Lalaro, Lautem District (regency), Tutuala Subdistrict, eastern East Timor[17]	AMNH R160135, holotype	Alcohol-preserved	2007	LR215681
Elseya intermedia Gray, 1872	North Australia, upper part of Victoria[18]	BMNH 1947.3.4.14, holotype	Shell with mummified tissues	*ca*. 1870	LR215682
Euchelymys subglobosa Krefft, 1876	Amama River, S. E. New Guinea[19]	MSNG CE 2320, holotype	Dry. mummified	*ca*. 1875	LR215683
Hydraspis australis Gray, 1841	Unknown[14]	BMNH 1947.3.4.36, holotype	Dry, mounted	*ca*. 1840	LR215684
Hydraspis victoriae Gray, 1842	Victoria River, north-west coast of New Holland[20]	BMNH 1947.3.5.95, lectotype (designated by Wells & Welling-ton)[13,]**	Shell with mummified tissues	*ca*. 1840	LR215685
Hydraspis victoriae Gray, 1842	Victoria River, north-west coast of New Holland[20]	BMNH 1947.3.5.96, paralectotype (desig-nated by Wells & Wellington)[13,]**	Shell with mummified tissues	*ca*. 1840	LR215686
Phrynops bellii Gray, 1844	Unknown[21]	OUM 8460, holotype	Mummified dry juvenile	*ca*. 1840	LR215687
Platemys novaeguineae Meyer, 1874	Neu-Guinea[22]	MTD 8222, holotype	Alcohol-preserved	*ca*. 1873	failed
Testudo longicollis Shaw, 1794	Australia, Australasia or New Holland[23]	BMNH 1947.3.5.86, holotype	Dry, mounted	*ca*. 1790	LR215688

* See discussion in Iverson *et al.*[24].

** The putative paralectotype is to be identified with the holotype of *Hydraspis victoriae* Gray, 1841, see discussion in text.

Table S2. Studied fresh material and GenBank data.

Taxon	Collection site	Lab code	Specimen reference*	Accession number	Source
Chelodina burrungandjii	Australia: Western Australia: King Edward River, Surveyors Pool (-14.671, 125.735)	Chbur671	UC 0671	KY776447	Present study
Chelodina canni	Australia: Northern Territory: Roper River, Sunday Creek (-16.118, 133.572)	Chcan657	UC 0657	KY776448	Present study
Chelodina colliei	Australia: Western Australia: Swan River, Perth (-31.950, 115.850)	Chcol227	UC 0227	KY776449	Present study
Chelodina expansa	Australia: Queensland: Fitzroy River, Moura (-24.603, 149.913)	—	AA032872	KY705230	Present study
Chelodina expansa	Australia: New South Wales: Murray River, Mungabareena (-36.093, 146.948)	Chexp175	UC 0175	KY776450	Present study
Chelodina longicollis	Australia: Australian Capital Territory (-35.190, 149.110)	—	AA045603	KJ713173	Zhang & Georges[25]
Chelodina mccordi	Indonesia: Roti Island (*ca.* -10.762, 123.123)	—	UC 0493	KY705231	Present study
Chelodina parkeri	Papua New Guinea: Fly River, Suki-Aramba Swamp (-8.245, 141.767)	—	AA042607	KY705232	Present study
Chelodina pritchardi	Papua New Guinea: Laloki River (-9.031, 146.869)	—	AA021711	KY705233	Present study
Chelodina oblonga sensu Thomson[26]	Australia: Northern Territory: Finnis River, Knuckies Lagoon (-12.426, 130.938)	Chrug313	UC 0313	KY776451	Present study
Chelodina oblonga sensu Thomson[26]	Unknown	—	—	HQ172157	Wang *et al.*[27]
Chelodina steindachneri	Australia: Western Australia: Millbillillie Station (-26.621, 120.330)	Chste238	UC 0238	KY776452	Present study
Elseya albagula	Australia: Queensland: Burnett River, Grays Waterhole (-25.536, 151.658)	Elalb183	UC 0183	KY776453	Present study
Elseya branderhorsti	Unknown	—	—	KC692461	Nie & Hu, unpublished
Elseya dentata	Australia: Northern Territory: Daly River, Oolloo Crossing (-14.071, 131.251)	Elden260	UC 0260	KY779843	Present study
Elseya dentata	Australia: Northern Territory: Victoria River, Victoria River Crossing (-15.633, 131.133)	Elden258	UC 0258	KY779842	Present study
Elseya flaviventralis	Australia: Northern Territory: South Alligator River, Pine Creek Crossing (-13.499, 132.471)	Elmag255	UC 0255	KY776454	Present study
Elusor macrurus	Australia: Queensland: Mary River	—	—	KU736930	Schmidt *et al.*[28]

Table S2. *Continued*.

Taxon	Collection site	Lab code	Specimen reference*	Accession number	Source
Elusor macrurus	Australia: Queensland: Mary River	Elmac219	UC 0219	KY857551	Present study
Emydura macquarii	Australia: New South Wales: Murray River, Mungabareena (-36.093, 146.948)	Emmac119	UC 0119	KY857552	Present study
"Emydura subglobosa"	Unknown	—	—	KC692462	Nie & Hou, unpublished
Emydura subglobosa	Australia: Northern Territory: Roper River, Mataranka Springs (-14.925, 133.134)	Emsub205	UC 0205	KY857553	Present study
Emydura tanybaraga	Australia: Queensland: Mitchell River	Emtan220	UC 0220	KY857559	Present study
Emydura victoriae	Australia: Northern Territory: Daly River, Oolloo Crossing (-14.071, 131.251)	Emvic245	UC 0245	KY857554	Present study
Myuchelys bellii	Australia: New South Wales: Gwydir River (-30.496, 151.133)	—	UC 0177	KY924930	Present study
Myuchelys georgesi	Australia: New South Wales: Bellinger River (-30.444, 152.622)	Mygeo131	UC 0131	KY857555	Present study
Myuchelys latisternum	Australia: New South Wales: Tweed River, Byangum (-28.355, 153.360)	Mylat127	UC 0127	KY857556	Present study
Myuchelys purvisi	Australia: New South Wales: Manning River (-31.733, 151.850)	Mypur130	UC 0130	KY883378	Present study
Pseudemydura umbrina	Australia: Western Australia: Ellen Brook Nature Reserve, Perth (-31.750, 116.033)	—	—	KY486272	Zhang *et al.*[29]
Rheodytes leukops	Australia: Queensland: Fitzroy River	Rhleu239	UC 0239	KY857558	Present study
South American taxa					
Chelus fimbriata	Unknown	—	—	HQ172156	Wang *et al.*[27]
Mesoclemmys hogei	Unknown	—	—	MF615513	Prosdocimi *et al.*, unpublished
Platemys platycephala	Unknown	—	—	KC692464	Ni & Hou, unpublished
Outgroup					
Pelomedusa variabilis	Unknown	—	—	AF039066	Zardoya & Meyer[30]

*Specimen reference provides a link to full data held in the Wildlife Tissue Collection at the University of Canberra.

Table S3. Evolutionary models for the 'FULL alignment' suggested by PARTITIONFINDER2 for the RAxML and MRBAYES analyses.

Alignment position	Partition	Model RAxML	Model MRBAYES
1–24	tRNA	GTR+I+G	GTR+I+G
25–1034	12S	GTR+I+G	GTR+I+G
1035–1110	tRNA	GTR+I+G	GTR+I+G
1111–2792	16S	GTR+G	GTR+G
2793–2873	tRNA	GTR+I+G	GTR+I+G
2874–3842\3	ND1_pos1	GTR+I+G	SYM+G
2875–3842\3	ND1_pos2	GTR+I+G	HKY+I+G
2876–3842\3	ND1_pos3	GTR+G	HKY+G
3843–4058	tRNA	GTR+I+G	GTR+I+G
4059–5100\3	ND2_pos1	GTR+I+G	GTR+I+G
4060–5100\3	ND2_pos2	GTR+I+G	GTR+I+G
4061–5100\3	ND2_pos3	GTR+I+G	HKY+I+G
5101–5500	tRNA	GTR+I+G	GTR+I+G
5501–7042\3	coxI_pos1	GTR+G	SYM+G
5502–7042\3	coxI_pos2	GTR+I+G	HKY+I
5503–7042\3	coxI_pos3	GTR+I+G	GTR+I+G
7043–7217	tRNA	GTR+I+G	GTR+I+G
7218–7904\3	coxII_pos1	GTR+G	SYM+G
7219–7904\3	coxII_pos2	GTR+I+G	HKY+I
7220–7904\3	coxII_pos3	GTR+G	GTR+G
7905–7984	tRNA	GTR+I+G	GTR+I+G
7985–8140\3	atp8_pos1	GTR+G	GTR+G
7986–8140\3	atp8_pos2	GTR+G	HKY+G
7987–8140\3	atp8_pos3	GTR+G	HKY+G
8141–8810\3	atp6_pos1	GTR+I+G	HKY+I+G
8142–8810\3	atp6_pos2	GTR+I+G	GTR+I+G
8143–8810\3	atp6_pos3	GTR+I+G	HKY+G

Alignment position	Partition	Model RAxML	Model MRBAYES
8811–9584\3	coxIII_pos1	GTR+I+G	K80+I+G
8812–9584\3	coxIII_pos2	GTR+I+G	HKY+I+G
8813–9584\3	coxIII_pos3	GTR+I+G	GTR+G
9585–9656	tRNA	GTR+I+G	GTR+I+G
9657–10001\3	ND3_pos1	GTR+I+G	HKY+G
9658–10001\3	ND3_pos2	GTR+G	HKY+G
9659–10001\3	ND3_pos3	GTR+G	HKY+G
10002–10073	tRNA	GTR+I+G	GTR+I+G
10074–10361\3	ND4L_pos1	GTR+G	HKY+G
10075–10361\3	ND4L_pos2	GTR+G	HKY+G
10076–10361\3	ND4L_pos3	GTR+G	GTR+G
10362–11732\3	ND4_pos1	GTR+I+G	GTR+I+G
10363–11732\3	ND4_pos2	GTR+I+G	GTR+I+G
10364–11732\3	ND4_pos3	GTR+G	GTR+G
11733–11948	tRNA	GTR+I+G	GTR+I+G
11949–13763\3	ND5_pos1	GTR+G	GTR+G
11950–13763\3	ND5_pos2	GTR+G	HKY+G
11951–13763\3	ND5_pos3	GTR+I+G	GTR+I+G
13764–14292\3	ND6_pos3	GTR+G	HKY+G
13765–14292\3	ND6_pos2	GTR+I+G	HKY+I+G
13766–14292\3	ND6_pos1	GTR+G	HKY+G
14293–14463	tRNA	GTR+I+G	GTR+I+G
14464–15603\3	cytb_pos1	GTR+G	GTR+G
14465–15603\3	cytb_pos2	GTR+G	GTR+G
14466–15603\3	cytb_pos3	GTR+G	GTR+G
15604–15753	tRNA	GTR+I+G	GTR+I+G
15754–16289	CR	GTR+G	GTR+G

Table S4. Evolutionary models for the 'CDS alignment' suggested by PARTITIONFINDER2 for the RAxML and MRBAYES analyses.

Alignment position	Partition	Model RAxML	Model MrBayes
1–969\3	ND1_pos1	GTR+I+G	SYM+G
2–970\3	ND1_pos2	GTR+I+G	HKY+I+G
3–971\3	ND1_pos3	GTR+G	HKY+G
970–2011\3	ND2_pos1	GTR+I+G	GTR+I+G
971–2011\3	ND2_pos2	GTR+I+G	GTR+I+G
972–2011\3	ND2_pos3	GTR+I+G	HKY+I+G
2012–3553\3	coxI_pos1	GTR+G	SYM+G
2013–3553\3	coxI_pos2	GTR+G	HKY+I
2014–3553\3	coxI_pos3	GTR+I+G	GTR+I+G
3554–4240\3	coxII_pos1	GTR+G	GTR+I
3555–4240\3	coxII_pos2	GTR+I+G	HKY+I
3556–4240\3	coxII_pos3	GTR+G	GTR+G
4241–4396\3	atp8_pos1	GTR+G	GTR+G
4242–4396\3	atp8_pos2	GTR+G	HKY+G
4243–4396\3	atp8_pos3	GTR+G	HKY+G
4397–5066\3	atp6_pos1	GTR+I+G	HKY+I+G
4398–5066\3	atp6_pos2	GTR+I+G	GTR+I+G
4399–5066\3	atp6_pos3	GTR+G	HKY+G
5067–5840\3	coxIII_pos1	GTR+I+G	K80+I+G
5068–5840\3	coxIII_pos2	GTR+I+G	HKY+I+G
5069–5840\3	coxIII_pos3	GTR+G	GTR+G
5841–6185\3	ND3_pos1	GTR+I+G	HKY+G
5842–6185\3	ND3_pos2	GTR+G	HKY+G
5843–6185\3	ND3_pos3	GTR+G	HKY+G
6186–6473\3	ND4L_pos1	GTR+G	HKY+G
6187–6473\3	ND4L_pos2	GTR+G	HKY+G
6188–6473\3	ND4L_pos3	GTR+G	GTR+G
6474–7844\3	ND4_pos1	GTR+I+G	GTR+I+G
6475–7844\3	ND4_pos2	GTR+I+G	GTR+I+G
6476–7844\3	ND4_pos3	GTR+I+G	GTR+G
7845–9659\3	ND5_pos1	GTR+G	GTR+G
7846–9659\3	ND5_pos2	GTR+I+G	HKY+I+G
7847–9659\3	ND5_pos3	GTR+I+G	GTR+I+G
9660–10188\3	ND6_pos3	GTR+G	HKY+G
9661–10188\3	ND6_pos2	GTR+I+G	HKY+I+G
9662–10188\3	ND6_pos1	GTR+G	HKY+G
10189–11328\3	cytb_pos1	GTR+G	GTR+G
10190–11328\3	cytb_pos2	GTR+G	GTR+G
10191–11328\3	cytb_pos3	GTR+G	GTR+G

Table S5. Uncorrected *p* distances of mitogenomes of Australasian chelid turtles based on 16,473 aligned sites.

		1	2	3	4	5	6	7	8	9	10	11	12	13	14	15	16	17	18	19	20	21	22	23	24
**1**	*Chelodina parkeri* KY705232	–																							
**2**	*Chelodina steindachneri* Chste238	0.1080	–																						
**3**	*Chelodina oblonga* holotype	0.1002	0.0653	–																					
**4**	*Chelodina colliei* Chcol227	0.1007	0.0653	0.0031	–																				
**5**	*Testudo longicollis* holotype	0.1074	0.0760	0.0649	0.0649	–																			
**6**	*Chelodina expansa* Chexp175	0.1080	0.0766	0.0657	0.0658	0.0132	–																		
**7**	*Chelodina pritchardi* KY705233	0.1104	0.0704	0.0699	0.0703	0.0435	0.0437	–																	
**8**	*Chelodina gunaleni* holotype	0.1102	0.0809	0.0710	0.0709	0.0403	0.0412	0.0318	–																
**9**	*Chelodina novaeguineaea* lectotype	0.1099	0.0797	0.0702	0.0700	0.0393	0.0400	0.0311	0.0137	–															
**10**	*Chelodina reimanni* paratype	0.1101	0.0797	0.0703	0.0702	0.0397	0.0402	0.0311	0.0139	0.0021	–														
**11**	*Chelodina reimanni* holotype	0.1102	0.0799	0.0705	0.0704	0.0399	0.0404	0.0313	0.0141	0.0022	0.0002	–													
**12**	*Chelodina mccordi* paratype	0.1085	0.0772	0.0699	0.0699	0.0388	0.0415	0.0458	0.0420	0.0421	0.0423	0.0425	–												
**13**	*Chelodina mccordi* KY705231	0.1084	0.0772	0.0698	0.0699	0.0387	0.0414	0.0458	0.0420	0.0420	0.0423	0.0425	0.0001	–											
**14**	*Chelodina mccordi roteensis* holotype	0.1081	0.0770	0.0697	0.0698	0.0386	0.0414	0.0455	0.0417	0.0418	0.0420	0.0422	0.0007	0.0006	–										
**15**	*Chelodina mccordi timorlestensis* holotype	0.1093	0.0782	0.0700	0.0701	0.0381	0.0407	0.0452	0.0409	0.0405	0.0407	0.0409	0.0101	0.0102	0.0102	–									
**16**	*Chelodina timorensis* holotype	0.1093	0.0783	0.0702	0.0703	0.0383	0.0409	0.0453	0.0411	0.0405	0.0408	0.0410	0.0103	0.0104	0.0104	0.0002	–								
**17**	*Chelodina burrungandjii* Chbur671	0.1104	0.0793	0.0717	0.0721	0.0395	0.0408	0.0457	0.0422	0.0432	0.0431	0.0433	0.0286	0.0285	0.0285	0.0277	0.0279	–							
**18**	*Chelodina canni* Chcan657	0.1076	0.0766	0.0689	0.0690	0.0366	0.0382	0.0435	0.0399	0.0398	0.0401	0.0401	0.0245	0.0244	0.0244	0.0242	0.0244	0.0236	–						
**19**	*Chelodina expansa* KY705230	0.1082	0.0770	0.0686	0.0686	0.0358	0.0373	0.0429	0.0385	0.0383	0.0385	0.0387	0.0238	0.0238	0.0238	0.0235	0.0236	0.0223	0.0140	–					
**20**	*Chelodina longicollis* KJ713173	0.1082	0.0766	0.0687	0.0687	0.0356	0.0371	0.0422	0.0379	0.0373	0.0376	0.0378	0.0230	0.0230	0.0230	0.0229	0.0231	0.0219	0.0133	0.0025	–				
**21**	*Chelodina kuchlingi holotype*	0.1037	0.0780	0.0667	0.0672	0.0615	0.0621	0.0658	0.0640	0.0620	0.0624	0.0626	0.0588	0.0588	0.0585	0.0572	0.0573	0.0612	0.0596	0.0585	0.0578	–			
**22**	*Chelodina oblonga* sensu Thomson Chrug313	0.1115	0.0895	0.0795	0.0793	0.0877	0.0877	0.0900	0.0924	0.0912	0.0915	0.0917	0.0915	0.0914	0.0911	0.0910	0.0912	0.0909	0.0898	0.0900	0.0896	0.0378	–		
**23**	*Chelodina oblonga* sensu Thomson HQ172157	0.1110	0.0878	0.0815	0.0812	0.0900	0.0904	0.0915	0.0941	0.0925	0.0924	0.0925	0.0913	0.0913	0.0910	0.0910	0.0912	0.0921	0.0908	0.0913	0.0910	0.0645	0.0571	–	
**24**	*Chelodina siebenrocki* holotype	0.1099	0.0855	0.0800	0.0798	0.0886	0.0887	0.0899	0.0921	0.0908	0.0907	0.0908	0.0897	0.0896	0.0894	0.0893	0.0895	0.0899	0.0887	0.0887	0.0885	0.0629	0.0553	0.0151	–
**25**	*Elseya dentata* Elden260	0.1808	0.1843	0.1836	0.1835	0.1832	0.1837	0.1837	0.1828	0.1823	0.1826	0.1827	0.1821	0.1821	0.1817	0.1826	0.1826	0.1841	0.1833	0.1835	0.1830	0.1773	0.1856	0.1865	0.1846
**26**	*Elseya intermedia* holotype	0.1806	0.1843	0.1841	0.1839	0.1828	0.1833	0.1836	0.1829	0.1820	0.1825	0.1826	0.1815	0.1815	0.1811	0.1825	0.1825	0.1844	0.1834	0.1834	0.1831	0.1771	0.1854	0.1862	0.1844
**27**	*Elseya dentata* Elden258	0.1806	0.1845	0.1843	0.1841	0.1829	0.1834	0.1837	0.1829	0.1820	0.1825	0.1826	0.1815	0.1815	0.1812	0.1825	0.1826	0.1843	0.1833	0.1834	0.1831	0.1774	0.1856	0.1862	0.1845
**28**	*Elseya branderhorsti* KC692461	0.1809	0.1844	0.1845	0.1840	0.1835	0.1840	0.1835	0.1833	0.1826	0.1827	0.1827	0.1819	0.1819	0.1817	0.1826	0.1826	0.1840	0.1830	0.1831	0.1827	0.1775	0.1850	0.1854	0.1835
**29**	*Elseya flaviventralis* Elmag255	0.1810	0.1840	0.1838	0.1835	0.1819	0.1825	0.1826	0.1819	0.1808	0.1812	0.1812	0.1803	0.1803	0.1801	0.1806	0.1806	0.1817	0.1814	0.1814	0.1811	0.1758	0.1835	0.1857	0.1840
**30**	*Elseya albagula* Elalb183	0.1828	0.1856	0.1834	0.1835	0.1846	0.1853	0.1850	0.1849	0.1846	0.1850	0.1850	0.1832	0.1831	0.1828	0.1843	0.1844	0.1857	0.1846	0.1856	0.1852	0.1765	0.1835	0.1862	0.1848
**31**	*Myuchelys georgesi* Mygeo131	0.1759	0.1803	0.1759	0.1760	0.1773	0.1793	0.1800	0.1801	0.1788	0.1789	0.1791	0.1777	0.1776	0.1771	0.1783	0.1784	0.1788	0.1780	0.1781	0.1779	0.1721	0.1831	0.1816	0.1810
**32**	*Myuchelys latisternum* Mylat127	0.1753	0.1788	0.1737	0.1742	0.1757	0.1759	0.1780	0.1779	0.1769	0.1770	0.1772	0.1768	0.1767	0.1763	0.1771	0.1772	0.1771	0.1767	0.1769	0.1769	0.1699	0.1791	0.1806	0.1784
**33**	*Myuchelys bellii* KY924930	0.1756	0.1801	0.1752	0.1755	0.1774	0.1783	0.1799	0.1800	0.1790	0.1791	0.1793	0.1790	0.1790	0.1785	0.1791	0.1792	0.1792	0.1788	0.1787	0.1788	0.1715	0.1806	0.1817	0.1797
**34**	*Phrynops bellii* holotype	0.1750	0.1792	0.1744	0.1746	0.1764	0.1774	0.1789	0.1792	0.1782	0.1783	0.1785	0.1780	0.1780	0.1774	0.1783	0.1783	0.1783	0.1778	0.1778	0.1779	0.1707	0.1798	0.1808	0.1789
**35**	*Hydraspis victoriae* paralectotype	0.1746	0.1785	0.1744	0.1746	0.1771	0.1769	0.1798	0.1794	0.1779	0.1782	0.1784	0.1786	0.1786	0.1782	0.1792	0.1792	0.1780	0.1786	0.1787	0.1784	0.1696	0.1793	0.1818	0.1802
**36**	*Hydraspis australis* holotype	0.1768	0.1807	0.1763	0.1765	0.1775	0.1780	0.1809	0.1801	0.1789	0.1792	0.1794	0.1793	0.1792	0.1788	0.1796	0.1797	0.1786	0.1791	0.1790	0.1788	0.1699	0.1810	0.1830	0.1816
**37**	*Emydura macquarii* Emmac119	0.1759	0.1805	0.1756	0.1758	0.1770	0.1773	0.1798	0.1792	0.1776	0.1778	0.1780	0.1782	0.1782	0.1775	0.1789	0.1789	0.1777	0.1780	0.1782	0.1778	0.1699	0.1809	0.1817	0.1800
**38**	*Hydraspis victoriae* lectotype	0.1749	0.1799	0.1750	0.1752	0.1765	0.1768	0.1792	0.1787	0.1770	0.1772	0.1774	0.1776	0.1776	0.1769	0.1784	0.1784	0.1772	0.1776	0.1778	0.1774	0.1693	0.1803	0.1810	0.1796
**39**	*Euchelymys subglobosa* holotype	0.1802	0.1831	0.1782	0.1783	0.1793	0.1795	0.1819	0.1816	0.1806	0.1809	0.1811	0.1805	0.1804	0.1801	0.1809	0.1808	0.1817	0.1806	0.1815	0.1810	0.1729	0.1834	0.1857	0.1846
**40**	*Emydura subglobosa* Emsub205	0.1788	0.1824	0.1759	0.1757	0.1775	0.1775	0.1804	0.1804	0.1789	0.1791	0.1793	0.1775	0.1775	0.1772	0.1785	0.1786	0.1793	0.1785	0.1792	0.1787	0.1718	0.1833	0.1838	0.1823
**41**	*'Emydura subglobosa'* KC692462	0.1822	0.1858	0.1815	0.1819	0.1827	0.1829	0.1858	0.1854	0.1848	0.1850	0.1852	0.1833	0.1833	0.1829	0.1832	0.1833	0.1845	0.1840	0.1842	0.1840	0.1761	0.1857	0.1875	0.1866
**42**	*Emydura tanybaraga* Emtan220	0.1823	0.1857	0.1815	0.1818	0.1825	0.1827	0.1856	0.1852	0.1845	0.1847	0.1849	0.1831	0.1831	0.1827	0.1830	0.1831	0.1843	0.1838	0.1840	0.1838	0.1759	0.1856	0.1875	0.1866
**43**	*Emydura victoriae* Emvic245	0.1825	0.1846	0.1815	0.1818	0.1822	0.1824	0.1852	0.1852	0.1841	0.1843	0.1845	0.1826	0.1825	0.1821	0.1825	0.1825	0.1837	0.1835	0.1836	0.1836	0.1753	0.1857	0.1867	0.1860
**44**	*Myuchelys purvisi* Mypur130	0.1777	0.1804	0.1783	0.1786	0.1773	0.1782	0.1778	0.1790	0.1785	0.1788	0.1789	0.1778	0.1778	0.1773	0.1779	0.1780	0.1774	0.1780	0.1773	0.1771	0.1725	0.1815	0.1828	0.1812
**45**	*Elusor macrurus* Elmac219	0.1766	0.1817	0.1806	0.1811	0.1794	0.1814	0.1815	0.1824	0.1813	0.1816	0.1818	0.1807	0.1807	0.1802	0.1811	0.1811	0.1810	0.1804	0.1811	0.1807	0.1732	0.1819	0.1830	0.1822
**46**	*Elusor macrurus* KU736930	0.1766	0.1817	0.1806	0.1811	0.1794	0.1814	0.1815	0.1824	0.1813	0.1816	0.1818	0.1807	0.1807	0.1802	0.1811	0.1811	0.1810	0.1804	0.1811	0.1807	0.1732	0.1819	0.1830	0.1822
**47**	*Rheodytes leukops* Rhleu239	0.1785	0.1819	0.1799	0.1798	0.1769	0.1777	0.1803	0.1798	0.1788	0.1790	0.1792	0.1787	0.1787	0.1783	0.1790	0.1790	0.1800	0.1785	0.1790	0.1790	0.1728	0.1806	0.1816	0.1788
**48**	*Pseudemydura umbrina* KY486272	0.1993	0.1963	0.1972	0.1970	0.1949	0.1963	0.1956	0.1956	0.1946	0.1952	0.1952	0.1954	0.1954	0.1952	0.1949	0.1950	0.1954	0.1942	0.1946	0.1946	0.1913	0.2010	0.1983	0.1963

Table S5. *Continued.*

		25	26	27	28	29	30	31	32	33	34	35	36	37	38	39	40	41	42	43	44	45	46	47	48
**25**	*Elseya dentata* Elden260	–																							
**26**	*Elseya intermedia* holotype	0.0120	–																						
**27**	*Elseya dentata* Elden258	0.0121	0.0003	–																					
**28**	*Elseya branderhorsti* KC692461	0.0234	0.0239	0.0240	–																				
**29**	*Elseya flaviventralis* Elmag255	0.0259	0.0263	0.0263	0.0204	–																			
**30**	*Elseya albagula* Elalb183	0.0670	0.0676	0.0677	0.0663	0.0660	–																		
**31**	*Myuchelys georgesi* Mygeo131	0.1011	0.1014	0.1016	0.1002	0.1003	0.0943	–																	
**32**	*Myuchelys latisternum* Mylat127	0.1005	0.1001	0.1004	0.0994	0.0981	0.0936	0.0494	–																
**33**	*Myuchelys bellii* KY924930	0.1015	0.1013	0.1015	0.1005	0.0995	0.0949	0.0514	0.0184	–															
**34**	*Phrynops bellii* holotype	0.1006	0.1002	0.1005	0.0998	0.0986	0.0942	0.0512	0.0181	0.0011	–														
**35**	*Hydraspis victoriae* paralectotype	0.1046	0.1043	0.1044	0.1030	0.1015	0.0971	0.0610	0.0577	0.0588	0.0585	–													
**36**	*Hydraspis australis* holotype	0.1062	0.1057	0.1059	0.1047	0.1031	0.0977	0.0619	0.0589	0.0604	0.0600	0.0068	–												
**37**	*Emydura macquarii* Emmac119	0.1035	0.1038	0.1040	0.1022	0.1010	0.0958	0.0604	0.0562	0.0576	0.0572	0.0232	0.0235	–											
**38**	*Hydraspis victoriae* lectotype	0.1031	0.1031	0.1033	0.1017	0.1007	0.0955	0.0598	0.0555	0.0570	0.0570	0.0231	0.0233	0.0002	–										
**39**	*Euchelymys subglobosa* holotype	0.1102	0.1107	0.1109	0.1086	0.1089	0.1015	0.0709	0.0669	0.0681	0.0677	0.0668	0.0671	0.0649	0.0646	–									
**40**	*Emydura subglobosa* Emsub205	0.1067	0.1072	0.1074	0.1060	0.1058	0.0991	0.0694	0.0650	0.0659	0.0657	0.0651	0.0652	0.0629	0.0625	0.0264	–								
**41**	'*Emydura subglobosa*' KC692462	0.1130	0.1137	0.1139	0.1121	0.1115	0.1057	0.0754	0.0726	0.0727	0.0723	0.0731	0.0735	0.0719	0.0715	0.0544	0.0527	–							
**42**	*Emydura tanybaraga* Emtan220	0.1128	0.1136	0.1137	0.1120	0.1114	0.1057	0.0752	0.0724	0.0726	0.0723	0.0729	0.0732	0.0717	0.0713	0.0540	0.0524	0.0008	–						
**43**	*Emydura victoriae* Emvic245	0.1114	0.1125	0.1126	0.1116	0.1110	0.1041	0.0745	0.0723	0.0722	0.0719	0.0720	0.0729	0.0715	0.0712	0.0538	0.0517	0.0119	0.0116	–					
**44**	*Myuchelys purvisi* Mypur130	0.1156	0.1149	0.1150	0.1153	0.1142	0.1095	0.0994	0.0985	0.0987	0.0984	0.1029	0.1044	0.1012	0.1007	0.1096	0.1069	0.1123	0.1121	0.1105	–				
**45**	*Elusor macrurus* Elmac219	0.1123	0.1111	0.1113	0.1111	0.1115	0.1072	0.0955	0.0941	0.0947	0.0941	0.0970	0.0985	0.0962	0.0956	0.1030	0.1016	0.1083	0.1080	0.1070	0.0795	–			
**46**	*Elusor macrurus* KU736930	0.1123	0.1111	0.1113	0.1111	0.1115	0.1072	0.0955	0.0941	0.0947	0.0941	0.0970	0.0985	0.0962	0.0956	0.1030	0.1016	0.1083	0.1080	0.1070	0.0795	0.0000	–		
**47**	*Rheodytes leukops* Rhleu239	0.1156	0.1160	0.1163	0.1163	0.1147	0.1115	0.1017	0.1009	0.0991	0.0991	0.1035	0.1053	0.1029	0.1025	0.1074	0.1049	0.1131	0.1130	0.1125	0.0975	0.0953	0.0953	–	
**48**	*Pseudemydura umbrina* KY486272	0.1888	0.1883	0.1883	0.1889	0.1875	0.1900	0.1844	0.1820	0.1816	0.1817	0.1811	0.1829	0.1816	0.1815	0.1868	0.1852	0.1880	0.1879	0.1871	0.1834	0.1849	0.1849	0.1867	–

Table S6. Information on DNA extraction and double-stranded DNA library (dsLib) build. Abbreviations: HT—holotype, LT—lectotype, PT—paratype, PLT—paralectotype.

Nominal taxon	Collection number	Type status	Tissue source for DNA extraction	Preserved since	Amount of tissue used for DNA extraction (mg)	DNA conc. Qubit HS (ng/µl)	Total amount of DNA (ng)	Input DNA for dsLib build (ng)
**Samples processed in aDNA laboratory**								
Chelodina kuchlingi Cann, 1997	WAM R29411	HT	EtOH-preserved muscle tissue from thigh	*ca*. 1970	7.1	0.306	30.6	8.568
Chelodina novaeguineae Boulenger, 1888	BMNH 1946.1.22.36	LT	Dry tissue from inside shell	*ca*. 1885	7.0	too low (<0.010)	<1.0	<0.280
Chelodina oblonga Gray, 1841	BMNH 1947.3.5.89	HT	Dry tissue from inside shell	*ca*. 1840	14.4	1.960	196.0	54.880
Chelodina reimanni Philippen & Großmann, 1990	MTD 29178	HT	EtOH-preserved muscle tissue from thigh	1990	18.0	0.212	21.2	5.936
Chelodina siebenrocki Werner, 1901	ZMB 16491	HT	EtOH-preserved muscle tissue from thigh	*ca*. 1900	7.5	too low (<0.010)	<1.0	<0.280
Elseya intermedia Gray, 1872	BMNH 1947.3.4.14	HT	Dry tissue from inguinal region	*ca*. 1870	23.3	2.500	250.0	70.000
Euchelymys subglobosa Krefft, 1876	CE 2320	HT	Dry skin	*ca*. 1875	29.9	4.140	414.0	115.920
Hydraspis australis Gray, 1841	BMNH 1947.3.4.36	HT	Dry tissue from inside shell	*ca*. 1840	35.1	0.164	16.4	4.592
Hydraspis victoriae Gray, 1842	BMNH 1947.3.5.95	LT*	Dry tissue from inguinal region	*ca*. 1840	6.2	0.394	39.4	11.032
Hydraspis victoriae Gray, 1842	BMNH 1947.3.5.96	PLT*	Dry tissue from inguinal region	*ca*. 1840	13.0	0.332	33.2	9.296
Phrynops bellii Gray, 1844	OUM 8460	HT	Dry tissue from inside shell	*ca*. 1840	33.8	1.380	138.0	38.640
Platemys novaeguineae Meyer, 1874	MTD 8222	HT	EtOH-preserved muscle tissue from thigh; specimen boiled during WWII attack	*ca*. 1873	26.4	too low (<0.010)	<1.0	<0.280
Testudo longicollis Shaw, 1792	BMNH 1947.3.5.86	HT	Dry tissue	*ca*. 1790	3.8	0.230	23.0	6.440

Table S6. *Continued.*

Nominal taxon	Collection number	Type status	Tissue source for DNA extraction	Preserved since	Amount of tissue used for DNA extraction (mg)	DNA conc. Qubit HS (ng/µl)	Total amount of DNA (ng)	Input DNA for dsLib build (ng)
**Samples processed in modern DNA laboratory**								
Chelodina gunaleni McCord & Joseph-Ouni, 2007	AMNH R160133	HT	EtOH-preserved muscle tissue from thigh	2007	n/a	0.594	59.4	16.632
Chelodina mccordi Rhodin, 1994	MCZ 176731	PT	EtOH-preserved muscle tissue from thigh	1994	n/a	4.680	468.0	131.040
Chelodina mccordi roteensis McCord, Joseph-Ouni & Hagen, 2007	AMNH R160132	HT	EtOH-preserved muscle tissue from thigh	2007	n/a	0.652	65.2	18.256
Chelodina mccordi timorlestensis Kuchling, Rhodin, Ibarrondo & Trainor, 2007	WAM 165888	HT	EtOH-preserved muscle tissue from thigh	2007	n/a	18.800	1880.0	526.400
Chelodina reimanni Philippen & Großmann, 1990	MTD 42828	PT	EtOH-preserved muscle tissue from thigh	1990	n/a	9.200	920.0	257.600
Chelodina timorensis McCord, Joseph-Ouni & Hagen 2007	AMNH R160135	HT	EtOH-preserved muscle tissue from thigh	2007	n/a	0.820	82.0	22.960

* The putative paralectotype is to be identified with the holotype of *H. victoriae* Gray, 1841, see discussion in text.

Table S7. Long range PCR primer pairs and internal Sanger sequencing primers used in this study, including their amplified product lengths and overlap.

**Long-range PCR primer pairs designed for bait-library preparation**		**Product size**	**Overlap**
based on a set of mt-genomes of all three genera including HQ172157			
Chelodina LR1 For	5´-ATGGCACTGAAGMTGCCAAGATG-3´	~8,700 bp	106 bp
Chelodina LR1 Rev	5´-TGAATAATAGCTACYGCTAGTTC-3´		
Chelodina LR2 For	5´-ATTACAGCAAAYYTAACAGCAGG-3´	~7,800 bp	
Chelodina LR2 Rev	5´-AAATACTATATGCCTATAARACC-3´		
Elseya/Emydura LR1 For	5´-ATGGCACTGAAGMYGCCAAGATG-3´	~8,700 bp	106 bp
Elseya/Emydura LR1 Rev	5´-TGRATAATGGCTACTGCTAGTTC-3´		
Elseya/Emydura LR2 For	5´-ATTACAGCAAAYYTAACAGCAGG-3´	~7,800 bp	
Elseya/Emydura LR2 Rev	5´-AAATACTATATGCCTATAARACC-3´		

**Internal Sanger sequencing primers for 12S and cyt *b***		**Source**
12S-L1091	5´-AAAAAGCTTCAAACTGGGATTAGATACCCCACTAT-3´	Kocher *et al.*[31]
mt-c-For2	5´-TGAGGVCARATATCATTYTGAG-3´	Fritz *et al.*[32]

Table S8. Long-range PCR data for bait library preparation.

Taxon	Collection number	Provenance	Annealing temperature for LR1/LR2	PCR cycles for LR1/LR2
Chelodina longicollis (Shaw, 1794)	MTD7251	Pet trade	55°C / 50°C	40 / 40
Elseya novaeguineae (Meyer, 1874)	MTD137	Pet trade	60°C / 50°C	35 / 35
Emydura australis (Gray, 1841)	MTD3933	Pet trade	60°C / 55°C	35 / 40

Table S9. The 184 deleted sited from the original alignment (16,473 bp), resulting in the 'FULL' alignment of 16,289 bp.

Locus	Cause	Positions excluded
atp8–atp6	10 bp overlap	14 bp (positions 8141–8154)
atp6–coxIII	7–8 bp overlap & frame shift	11 bp (positions 8825–8835)
ND3	Unknown frame shift mechanism	4 bp (position 9814–9817)
ND3	Unknown frame shift mechanism	1 bp (position 9857)
ND4L	Unknown frame shift mechanism	1 bp (position 10197)
ND4L	Unknown frame shift mechanism	1 bp (position 10363)
ND4L–ND4	7 bp overlap	11 bp (positions 10394–10404)
ND5–ND6	Unalignable non-coding region	141 bp (positions 13807–13947)

Table S10. Read information of sequenced type specimens with a mapping stringency of two allowed mismatches.

Nominal taxon	Raw reads	Merged reads (min. length 35 bp) without duplicates	% of raw reads	Readpool for assembly	Assembled reads	Average coverage	Average length of assembled reads in bp	MITObim iterations
**Samples processed in aDNA laboratory**								
Chelodina kuchlingi Cann, 1997	1,585,505	312,453	19.7	312,453	3,653	18	69	5
Chelodina novaeguineae Boulenger, 1888	1,340,173	475,633	35.,5	50,000	23,539	94	65	2
Chelodina oblonga Gray, 1841	1,468,609	905,238	61.6	50,000	28,728	138	78	3
Chelodina reimanni Philippen & Großmann, 1990	1,450,455	725,247	50.0	50,000	14,763	104	115	2
Chelodina siebenrocki Werner, 1901	3,497,952	423,990	12.1	423,990	13,248	80	97	5
Elseya intermedia Gray, 1872	1,591,120	1,160,747	73.0	30,000	22,437	146	107	4
Euchelymys subglobosa Krefft, 1876	1,360,169	1,042,845	76.7	30,000	21,975	160	120	4
Hydraspis australis Gray, 1841	1,462,815	1,063,969	72.7	30,000	22,402	142	104	4
Hydraspis victoriae Gray, 1842	1,232,106	810,984	65.8	50,000	26,493	140	87	4
Hydraspis victoriae Gray, 1842	1,400,179	372,030	26.6	50,000	18,830	83	71	6
Phrynops bellii Gray, 1844	1,334,864	464,092	34.8	50,000	14,724	88	97	4
Platemys novaeguineae Meyer, 1874	325,977	21,797	6.7	21,797	381	5	86	4
Testudo longicollis Shaw, 1792	1,308,995	383,199	29.3	383,199	23,902	91	62	3
**Samples processed in modern DNA laboratory**								
Chelodina gunaleni McCord & Joseph-Ouni, 2007	1,319,062	1,160,757	88.0	100,000	15,486	97	101	3
Chelodina mccordi Rhodin, 1994	1,479,988	1,137,594	76.9	100,000	17,660	85	60	2
Chelodina mccordi roteensis McCord, Joseph-Ouni & Hagen, 2007	1,499,959	1,079,959	71.9	30,000	19,169	138	119	2
Chelodina mccordi timorlestensis Kuchling, Rhodin, Ibarrondo & Trainor, 2007	1,568,861	1,180,767	75.3	30,000	20,663	118	93	2
Chelodina reimanni Philippen & Großmann, 1990	1,499,629	1,241,958	82.8	50,000	28,038	195	115	2
Chelodina timorensis McCord, Joseph-Ouni & Hagen 2007	1,465,038	1,159,918	79.2	30,000	9,935	71	114	2

Table S10. *Continued.*

Nominal taxon	Raw reads	Merged reads (min. length 35 bp) without duplicates	% of raw reads	Readpool for assembly	Assembled reads	Average coverage	Average length of assembled reads in bp	MITObim iterations
**Extraction and library blanks**								
Extraction blank aDNA	1,157,825	28,401	2.5	28,401	391	5	77	2
Extraction blank modern DNA	1,063,753	26,486	2.5	26,486	646	6	83	4
Library blank aDNA	952,184	13,472	1.4	13,472	498	5	79	2
Library blank modern DNA	1,058,561	16,701	1.6	16,701	454	5	77	2

References

1 Meyer, M. & Kircher, M. Illumina sequencing library preparation for highly multiplexed target capture and sequencing. *Cold Spring Harb. Protoc*. **2010**, pdb.prot5448 (2010).

2 Fortes, G. G. & Paijmans, J. L. A. Analysis of whole mitogenomes from ancient samples in *Whole Genome Amplification. Methods in Molecular Biology, 1347* (ed Kroneis, T.), 179–195 (Humana Press, 2015).

3 Maricic, T., Whitten, M. & Pääbo, S. Multiplexed DNA sequence capture of mitochondrial genomes using PCR products. *PLoS ONE* **5**, e14004 (2010).

4 Horn, S. Target enrichment via DNA hybridization capture in *Ancient DNA: Methods and Protocols. Methods in Molecular Biology, 840* (eds Shapiro, B. & Hofreiter, M.), 177–188 (Springer, 2012).

5 Fritz, U., Gemel, R., Kehlmaier, C., Vamberger, M. & Praschag, P. Phylogeography of the Asian softshell turtle *Amyda cartilaginea* (Boddaert, 1770): evidence for a species complex. *Vertebr. Zool*. **64**, 229–243 (2014).

6 Kumar, S., Stecher, G. & Tamura, K. MEGA7: Molecular Evolutionary Genetics Analysis version 7.0 for bigger datasets. *Mol. Biol. Evol.* **33**, 1870–1874 (2016).

7 McCord, W. P. & Joseph-Ouni, M. A new species of *Chelodina* (Testudines: Chelidae) from southwestern New Guinea (Papua, Indonesia). *Reptilia (GB)* **52**, 47–52 (2007).

8 Cann, J. Kuchling's long-neck Turtle. *Monitor* **9**, 31–32, 41–44 (1997).

9 Rhodin, A. G. J. Chelid turtles of the Australasian Archipelago: II. A new species of *Chelodina* from Roti Island, Indonesia. *Breviora* **498**, 1–31 (1994).

10 McCord, W. P., Joseph-Ouni, M. & Hagen, C. A new subspecies of *Chelodina mccordi* (Testudines: Chelidae) from eastern Rote Island, Indonesia. *Reptilia (GB)* **52**:58–61 (2007).

[11] Kuchling, G., Rhodin, A. G. J., Ibarrondo, B. R. & Trainor, C. R. A new subspecies of the snakeneck turtle *Chelodina mccordi* from Timor-Leste (East Timor) (Testudines: Chelidae). *Chelon. Conserv. Biol*. **6**, 213–222 (2007).
[12] Boulenger, G. A. On the chelydoid chelonians of New Guinea. *Ann. Mus. Civ. Stor. Nat. Genova (Ser. 2)* **6**, 449–452 (1888).
[13] Wells, R. W. & Wellington, C. R. A classification of the Amphibia and Reptilia of Australia. *Austral. J. Herpetol., Suppl. Ser.* **1**, 1–61 (1985).
[14] Gray, J. E. A catalogue of the species of reptiles and amphibia hitherto described as inhabiting Australia, with a description of some new species from Western Australia, and some remarks on their geographical distribution in *Journals of Two Expeditions of Discovery in Northwest and Western Australia, Vol. 2, Appendix E* (ed Grey, G.), 422–449 (T. and W. Boone, 1841).
[15] Philippen, H.-D. & Großmann, P. Eine neue Schlangenhalsschildkröte von Neuguinea: *Chelodina reimanni* sp. n. (Reptilia, Testudines, Pleurodira: Chelidae). *Zool. Abh.* **46**, 95–102 (1990).
[16] Werner, F. Ueber Reptilien und Batrachier aus Ecuador und Neu-Guinea. *Verh. Zool.-Bot. Ges. Wien* **51**, 593–603 (1901).
[17] McCord, W. P., Joseph-Ouni, M. & Hagen, C. A new species of *Chelodina* (Testudines: Chelidae) from eastern Timor Island (East Timor). *Reptilia (GB)* **52**, 53–57 (2007).
[18] Gray, J. E. *Appendix to the Catalogue of Shield Reptiles in the Collection of the British Museum. Part I. Testudinata (Tortoises)* (Trustees of the British Museum, 1872).
[19] Krefft, G. Notes on Australian animals in New Guinea with description of a new species of fresh water tortoise belonging to the genus *Euchelymys* (Gray). *Ann. Mus. Civ. Stor. Nat. Giacomo Doria (Ser. 1)* **8**, 390–394 (1876).
[20] Gray, J. E. Description of some hitherto unrecorded species of Australian reptiles and batrachians. *Zool. Misc*. **2**, 51–57 (1842).
[21] Gray, J. E. *Catalogue of the Tortoises, Crocodiles, and Amphisbaenians in the Collection of the British Museum* (Trustees of the British Museum, 1844).
[22] Meyer, A. B. *Platemys novaeguineae* sp. nov. Eine Mittheilung von Hrn. Adolf Bernhard Meyer über die von ihm auf Neu-Guinea und den Inseln Jobi, Mysore und Mafoor im Jahre 1873 gesammelten Amphibien. *Monatsber. Königl. Preuß. Akad. Wiss. Berlin* **39**, 128–140 (1874).
[23] Shaw, G. *Zoology of New Holland, Vol. I* (J. Davis, 1794).
[24] Iverson, J. B., Thomson, S. A. & Georges, A. The validity of the taxonomic changes for turtles proposed by Wells and Wellington. *J. Herpetol*. **35**, 361–368 (2001).
[25] Zhang, X. & Georges, A. A complete mitochondrial genome sequence for the Australian turtle, *Chelodina longicollis*, obtained using 454-pyrosequencing. *Conserv. Genet. Resour*. **6**, 555–557 (2014).
[26] Thomson, S. The identification of the holotype of *Chelodina oblonga* (Testudinata: Chelidae) with a discussion of taxonomic implications. *Chelon. Conserv. Biol*. **3**, 745–749 (2000).
[27] Wang, L. *et al*. The complete mitochondrial genome sequences of *Chelodina rugosa* and *Chelus fimbriata* (Pleurodira: Chelidae): implications of a common absence of initiation sites (OL) in pleurodiran turtles. *Mol. Biol. Rep*. **39**, 2097–2107 (2012).
[28] Schmidt, D. J., Brockett, B., Espinoza, T., Connell, M. & Hughes, J. Complete mitochondrial genome of the endangered Mary River turtle (*Elusor macrurus*) and low mtDNA variation across the species. *Austral. J. Zool*. **64**, 117–121 (2016).
[29] Zhang, X., Unmack, P. J., Kuchling, G., Wang, Y. & Georges, A. Resolution of the enigmatic phylogenetic relationship of the critically endangered western swamp tortoise *Pseudemydura umbrina* (Pleurodira: Chelidae) using a complete mitochondrial genome. *Mol. Phylogenet. Evol*. **115**, 58–61 (2017).
[30] Zardoya, R. & Meyer, A. Complete mitochondrial genome suggests diapsid affinities of turtles. *Proc. Natl. Acad. Sci. USA* **95**, 14226–14321 (1998).

[31] Kocher, T. D. *et al*. Dynamics of mitochondrial DNA evolution in mammals: amplification and sequencing with conserved primers. *Proc. Natl. Acad. Sci. USA* **86**, 6196–6200 (1989).
[32] Fritz, U. *et al*. A rangewide phylogeography of Hermann’s tortoise, *Testudo hermanni* (Reptilia: Testudines: Testudinidae): implications for taxonomy. *Zool. Scr*. **35**, 531–543 (2006).
